# Human T Cell Leukemia Virus Reactivation with Progression of Adult T-Cell Leukemia-Lymphoma

**DOI:** 10.1371/journal.pone.0004420

**Published:** 2009-02-10

**Authors:** Lee Ratner, William Harrington, Xuan Feng, Christian Grant, Steve Jacobson, Ariela Noy, Joseph Sparano, Jeannette Lee, Richard Ambinder, Nancy Campbell, Michael Lairmore, for the AIDS Malignancy Consortium

**Affiliations:** 1 Division of Oncology, Washington University School of Medicine, St Louis, Missouri, United States of America; 2 University of Miami School of Medicine, Miami, Florida, United States of America; 3 Neuroimmunology Branch, National Institute of Neurological Disorders and Stroke, Bethesda, Maryland, United States of America; 4 Lymphoma Service, Memorial Sloan Kettering Cancer Center, New York, New York, United States of America; 5 Department of Oncology, Albert Einstein College of Medicine, Bronx, New York, United States of America; 6 Division of Hematology/Oncology, University of Alabama School of Medicine, Birmingham, Alabama, United States of America; 7 Department of Oncology, Johns Hopkins University Medical Institutions, Baltimore, Maryland, United States of America; 8 Center for Retrovirus Research, The Ohio State University College of Veterinary Medicine, Columbus, Ohio, United States of America; University of Sao Paulo, Brazil

## Abstract

**Background:**

Human T-cell leukemia virus-associated adult T-cell leukemia-lymphoma (ATLL) has a very poor prognosis, despite trials of a variety of different treatment regimens. Virus expression has been reported to be limited or absent when ATLL is diagnosed, and this has suggested that secondary genetic or epigenetic changes are important in disease pathogenesis.

**Methods and Findings:**

We prospectively investigated combination chemotherapy followed by antiretroviral therapy for this disorder. Nineteen patients were prospectively enrolled between 2002 and 2006 at five medical centers in a phase II clinical trial of infusional chemotherapy with etoposide, doxorubicin, and vincristine, daily prednisone, and bolus cyclophosphamide (EPOCH) given for two to six cycles until maximal clinical response, and followed by antiviral therapy with daily zidovudine, lamivudine, and alpha interferon-2a for up to one year. Seven patients were on study for less than one month due to progressive disease or chemotherapy toxicity. Eleven patients achieved an objective response with median duration of response of thirteen months, and two complete remissions. During chemotherapy induction, viral RNA expression increased (median 190-fold), and virus replication occurred, coincident with development of disease progression.

**Conclusions:**

EPOCH chemotherapy followed by antiretroviral therapy is an active therapeutic regimen for adult T-cell leukemia-lymphoma, but viral reactivation during induction chemotherapy may contribute to treatment failure. Alternative therapies are sorely needed in this disease that simultaneously prevent virus expression, and are cytocidal for malignant cells.

**Trial Registration:**

ClinicalTrials.gov NCT00041327

## Introduction

Human T-cell leukemia virus type 1 (HTLV-1) is a member of the deltaretrovirus family [1]. Infections are prevalent in southern Japan, the Caribbean Islands, parts of Central and South America, the Middle East, and Africa, where 10–15% of the population is infected [2]. In the United States, 0.025% of volunteer blood donors are infected with HTLV-1, or the closely related retrovirus, HTLV-2. Infections by either virus are common among intravenous drug abusers.

HTLV-1 includes *gag, pro, pol,* and *env* genes, encoding the capsid of the virion, the viral protease, the viral reverse transcriptase and integrase enzymes, and the glycoprotein required for viral entry [1]. Several regulatory genes are critical for virus replication, spread, and pathology, especially *tax* which is capable of immortalizing lymphoid cells in culture and in mouse model systems. Tax-mediated transcriptional activation of nuclear factor κB (NFκB)-target genes is critical for resistance to apoptotic stimuli [2]. Tax also has an important role in T cell activation, proliferation, and genetic instability, factors critically important in leukogenesis [1].

Two to five percent of HTLV-1 infected patients develop myelopathy or a lymphoid malignancy, designated adult T-cell leukemia lymphoma (ATLL) [4], [5]. ATLL is classified as smoldering in about 5% of cases, chronic in 15% of cases, acute or leukemic in 60% of cases, and lymphomatous ATLL in 20% of cases [6]. Whereas smoldering and chronic ATLL are associated with median survivals of 5 and 2 yrs, respectively, the median survival of patients with leukemic and lymphomatous forms of ATLL is 0.5–2.0 yrs. ATLL is usually a malignancy of CD4+ T regulatory cells, although suppressor activity may be lacking, and occasional examples of CD8+ lymphoid malignancy have also been described [7]–[10]. ATLL is classified in a separate category in the Revised European American Lymphoma Classification, and as a peripheral T cell lymphoma in the World Health Organization classification [11], [12]. ATLL is characterized by frequent blood and bone marrow involvement, hypercalcemia, and lytic bone lesions [4].

HTLV-1 is uniformly associated with ATLL, as determined by antibody or nucleic acid assays, and clonality of HTLV-1 in tumor cells [1]. Nevertheless, viral expression is limited or absent when patients present with ATLL [1]. It has been hypothesized that Tax, and perhaps other HTLV-1 genes, are critical for initiation of the T-cell malignancy, but secondary genetic or epigenetic changes are required for disease progression.

Treatment approaches, with variable levels of success for ATLL have included various chemotherapy regimens, a combination of zidovudine and interferon alpha-2a, antibody or antibody-radioconjugate therapy, stem cell transplantation, or targeted approaches with bortezomib or arsenic trioxide [4], [5], [13].

The objectives of the current trial were to assess the efficacy of EPOCH chemotherapy followed by antiretroviral therapy in patients with the acute forms of ATLL, and evaluate the effects of therapy on HTLV-1 DNA and RNA load. The EPOCH regimen was chosen based on demonstrated activity in uninfected individuals with refractory B-cell non-Hodgkin's lymphomas, and in HIV-associated lymphomas [14], [15]. The antiretroviral therapy with interferon and zidovudine was selected based on demonstrated activity in the leukemic variant of ATLL [16], [17]. The addition of lamivudine to the antiretroviral therapy regimen was based on reported activity in HTLV-1 associated myelopathy [18]. Sequential, rather than concurrent therapy was chosen to avoid synergistic toxicity of the chemo- and anti-viral therapies.

## Methods

### Patient Selection

Inclusion criteria for the study were histologically or cytologically documented ATLL, with CD3+ tumors, documented HTLV-1 infection, evaluable or measurable disease, and adequate hematologic (absolute neutrophil count; ANC>1000, platelet count>75,000/mm^3^), hepatic (biliruin<2.0, transaminases<7 times upper limit of normal), and renal function (creatinine<2.0) unless abnormalities were due to ATLL involvement (Protocol S1). The study included patients with a Karnofsky performance score of at least 50%, at least 18 yrs of age who signed the informed consent, and allowed patients with prior treatment for ATLL. Exclusion criteria were active opportunistic infection requiring therapy, concurrent malignancy other than *in situ* cervical cancer or non-metastatic, non-melanomatous skin cancer, untreated thyroid disease, autoimmune disease, uncontrolled psychiatric disease, pregnancy or breastfeeding. Patients at any one of 50 different sites in the United States were eligibile for participation. Institutional review board approval was obtained at all enrolling sites, which included Memorial Sloan Kettering Cancer Center, New York, NY; Albert Einstein College of Medicine, University of Miami School of Medicine, Johns Hopkins University Medical Institutions, and Massachusetts General Hospital, as well as the coordinating site, Washington University School of Medicine. Written informed consent was obtained from all participants in the study.

### Treatment

**Figure 1:**
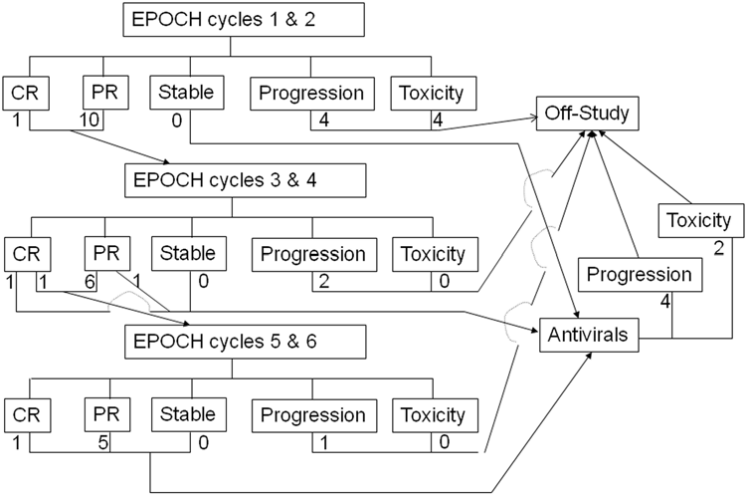
Treatment Schema. Subjects were treated with EPOCH chemotherapy for cycles 1 and 2 and then responses assessed. Patients with complete remissions (CR) or partial remissions (PR), received cycles 3 and 4, whereas patients with stable disease received antiviral therapy. Similar treatment assignments were applicable after cycles 3 and 4, with patients in CR or PR receiving cycles 5 and 6 EPOCH therapy, except for one patient who was in CR after cycles and 1 and 2, and remained in CR after cycles 3 and 4, and one patient in PR after cycles 1 and 2 and achieved “best response”, i.e. PR after cycles 3 and 4, who then received antiviral therapy. Subjects with stable disease, PR, or CR after 6 cycles of therapy then received antiviral therapy. Subjects with disease progression or toxicity during any stage of treatment, were taken off the treatment protocol, and were eligible to receive non-protocol therapies.

EPOCH therapy was administered as follows: etoposide 50 mg/m^2^/d, vincristine 0.4 mg/m^2^/d, and doxorubicin 10 mg/m^2^/d each given as a continuous 96 hr infusion on days 1–4, cyclophosphamide 750 mg/m^2^ given IV on day 5, and prednisone 60 mg/m^2^ given orally on days 1–5. Chemotherapy was administered on a 21–28 days cycle for a minimum of 2 cycles beyond best response and a maximum of six cycles (Fig 1). In contrast to the intra-patient dose modification in the NIH EPOCH regimen, no dose adjustments were planned based on the previous cycle's nadir blood counts. Responses were evaluated by standard criteria using clinical tumor measurements, peripheral blood absolute lymphocyte measurements, bone marrow biopsies, and CT scans [19]. “Best response” was defined as the response achieved when one or more additional cycles of chemotherapy were given and no additional tumor shrinkage was noted. For patients with stable disease or progressive disease, no additional chemotherapy was given. For patients with partial or complete remission, two additional cycles of chemotherapy were given, but no more than six cycles of chemotherapy. G-CSF was given at a dose of 5 ug/kg/d subcutaneously daily beginning 24 hrs after the administration of prednisone for 10 days beginning on day 6 or until ANC recovered to at least 4000 cells/mm^3^. Prophylaxis for Pneumocystis pneumonia was provided with co-trimoxazole, dapsone, or inhaled pentamidine. Allopurinol was recommended for seven days during the first cycle for patients with absolute lymphocyte count>50,000 or LDH>500 U/dl. Central nervous system prophylaxis was at the discretion of the investigator with either 50 mg cytosine arabinoside or 12 mg of intrathecal methotrexate administered on days 1, 8, 15, and 22 of the first chemotherapy cycle. One month after the last cycle of chemotherapy, antiretroviral therapy was initiated, which included zidovudine 300 mg bid plus lamivudine 150 mg bid (separately or as Combivir®) and interferon alpha-2a 9 mU subcutaneously per day for 1 yr.

Baseline evaluations included HTLV-1 serology, confirmatory immunoblot or PCR, hematology, blood chemistries, T-cell subsets, serum pregnancy test if appropriate, CT or MRI scans of the chest abdomen, and pelvis, bone marrow aspirate and biopsy, and lumbar puncture, HTLV-1 viral DNA and RNA. Evaluations during chemotherapy included blood chemistries and hematology with each cycle, and restaging CT and HTLV-1 viral DNA and RNA at the beginning of each odd numbered cycle and every 3 mos of antiretroviral therapy. Bone marrow biopsy was repeated at the beginning of each odd numbered cycle if positive at initiation of therapy.

Chemotherapy was delayed one week if ANC<1000 or platelets<75,000, and reduced if still depressed at that time. Reductions in doxorubicin and vincristine doses were prescribed if bilirubin was elevated. Doxorubicin was discontinued for congestive heart failure, and vincristine was discontinued for grade 3 or greater neurotoxicity. Interferon was discontinued and restarted at 5 mU/d if there was grade 3 fever, fatigue, anorexia, or mood disturbance. Interferon, zidovudine, and lamivudine were discontinued and restarted at a lower dose of interferon but full dose of combivir after recovering from grade 3 anemia, neutropenia, thrombocytopenia, or transaminase elevation. For recurrence of these toxicities, or persistent toxicities for more than thirty days, or other grade 4 toxicities, antiviral medications were permanently discontinued.

### Endpoints and Statistics

Patients were withdrawn from study if they developed a life threatening infection, chemotherapy was delayed for more than six weeks, severe toxicities developed, or progressive ATLL developed after two cycles of chemotherapy.

The target sample size was 29 subjects, but the study was closed after enrollment of 19 subjects due to slow accrual. The target sample size was based on a two-stage test of the null hypothesis that the overall response rate was <10%, against the alternative hypothesis that it was >30%, at the one-sided significance level of 5% and a power of 80% [20]. There were 10 patients in the first stage, and at least two objective responses were required to proceed with enrollment.

Binomial proportions and 95% confidence intervals were used to estimate response rates to therapy. Logistic regression analyses were used to evaluate baseline characteristics and other covariates with response. The Kaplan-Meier method was used to evaluate the response duration and overall survival. Analysis of variance was used to evaluate the effects of treatment and time on the viral DNA load measurements, and measurements of viral transcripts. A paired two-tailed t test was used to assess viral RNA and DNA values after logarithmic conversion at initiation of treatment compared to time when polymerase changes were noted.

### Viral DNA and RNA Measurements and Sequence Analyses

The HTLV-1 DNA assay, performed with peripheral blood mononuclear cells (PBMCs), prepared in a BSL3 facility, measured the number of copies of integrated or unintegrated viral genome using ABI PRISM 7700 sequence detector (Perkin Elmer/Applied Biosystems) with primers in pX [21]. The assay was standardized by measuring the number of copies of β-actin DNA, and performed in triplicate. The amount of HTLV-1 proviral DNA was calculated as copy number of HTLV-1 per 100 PBMC = [(copy number of px)/(copy number of β-actin/2)]×100.

The HTLV-1 RNA assay was performed with PBMCs. RNA extraction, complementary DNA (cDNA) synthesis, and real time PCR were performed with primers designed for amplification of HTLV-1 *tax* cDNA, and human housekeeping gene hypoxanthine ribosyl transferase (*hprt*) for internal calibration [22]. Standard curves were generated using cDNA from MT-2 cells, and all assays done in duplicate, with correlation values of standards more than 99%. The relative *tax* mRNA load was calculated by the following formula: HTLV-1 *tax* mRNA load = {(value of *tax*)/(value of *hprt*)}×10,000.

For DNA sequence analysis, genomic DNA was isolated from PBMCs using the Qiagen QIAamp DNA blood kit. PCR was carried out to amplify the *pol* gene (nt 2625–3195) encoding aa. 41-219, followed by automated DNA sequencing with Big Dye terminator V3.1.

## Results

### Patient Characteristics

Nineteen patients with CD4+ HTLV-1+ acute or lymphoma ATLL were enrolled between October, 2002 and February, 2006 at 5 medical centers in the United States (see Checklist S1). Characteristics of the patients at baseline and during their course of therapy are shown in Table 1. At baseline, an elevated absolute lymphocyte count was seen in 6 patients (range 400–127,000/cu mm), hypercalcemia in 1 patient, and stage III–IV disease in 16 of 18 patients; in one patient, staging was not completed for medical reasons. The LDH was elevated in 15 patients, performance status was 2–4 for 9 patients, age >60 yrs for 6 of 19 patients, and involvement of 2 or more extranodal sites in 5 of 18 patients. The revised International Prognostic Index score was 1–2 in 7 patients, and 3–5 in 11 patients [23].

**Table 1:** Patient Characteristics

Subject #	ALC	Stage	PS2-4	LDH elevation	Age>60	>2 extranodal	IPI	Best Response
1	2400	IV	yes	yes	yes	no	4	NE-sepsis
2	1400	III	yes	yes	no	yes	4	NE-toxicity
3	17200	IV	yes	yes	no	yes	4	NE-toxicity
4	1900	IV	yes	yes	no	no	3	NE-toxicity
5	1600	II	no	yes	yes	no	2	NE-PD
6	127000	IV	no	yes	no	yes	3	NE-PD
7	11700	ND	yes	no	yes	no	ND	NE-PD
8	2000	IV	no	no	no	no	1	PD
9	66000	IV	yes	yes	no	no	3	PR
10	17000	III	no	no	no	no	1	PR
11	500	IV	no	no	no	yes	2	PR
12	400	I	no	yes	no	no	2	PR
13	5400	IV	no	yes	yes	no	3	PR
14	500	IV	yes	yes	yes	no	4	PR
15	2300	IV	no	yes	no	no	2	PR
16	1800	III	no	yes	no	no	2	PR
17	1100	IV	yes	yes	no	yes	4	PR
18	1400	IV	no	yes	yes	no	3	CR
19	1500	IV	yes	yes	no	no	3	CR

Key: ALC, absolute lymphocyte count; PS, performance status (ECOG);

LDH, lactate dehydrogenase; IPI, international prognostic index; ND, not done;

CR, complete remission; NE, not evaluable; PR, partial remission; PD, progressive disease

Treatment was initiated with EPOCH chemotherapy, with a 4 day continuous infusion of etoposide, vincristine, and doxorubicin, day 5 cyclophosphamide, 5 days of prednisone, followed by 10 days of G-CSF. Five patients received 1 cycle of chemotherapy, 7 patients received 2–4 cycles of chemotherapy, and 7 patients received 5–6 cycles of chemotherapy (Fig 1). After completion of chemotherapy, 6 patients who had not manifested ATLL progression received antiviral therapy for a median of 1.5 mos (average of 3.4 mos, range 1–9 months); 2 eligible patients declined antiviral therapy.

Seven patients were non-evaluable, since they were on study for less than one month. This includes three patients with progressive disease, one patient each who died during the first cycle with fungal sepsis, cardiotoxicity, respiratory failure, or tumor lysis syndrome (Table 1).

### Viral RNA Expression

Levels of viral RNA and DNA were determined in PBMCs in 43 samples from these subjects, by real time PCR and RT-PCR assays (Supplementary Table S1). Viral DNA measurements were not predictive of response (not shown)

Viral RNA levels were normalized for levels of *hprt* mRNA (Supplementary Table S1). At diagnosis, viral RNA was undetectable in 6 subjects, expressed at 1.6-24 copies per 10^4^ copies of hprt RNA in 6 subjects, and at 180-283,000 copies per 10^4^ copies of *hprt* RNA in 3 subjects. Multiple samples were available for viral RNA measurements in 12 patients during their treatment course (Supplementary Table S1). In three subjects, no changes in viral RNA were detected (subjects 3, 10, 11). In two subjects, variable levels of viral RNA were seen during the course of treatment, variably low levels in subject 19 (0.6-55 copies per 10^4^ copies of hprt RNA), and variably high levels in subject 17 (2250-14,000 copies per 10^4^ copies of hprt RNA). In 6 subjects increasing viral RNA levels were found during the course of therapy (median 190-fold increase, range 11.8-97,800 fold increase; subjects 8, 9, 12, 13, 14, 15). In subject 16, decreasing viral RNA levels were seen over the course of therapy.

**Figure 2:**
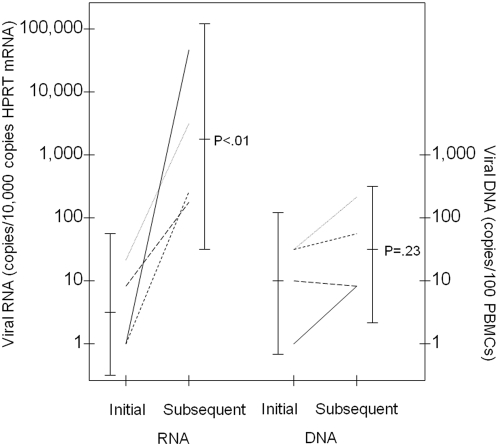
Viral RNA Changes with Polymerase Changes. The figure shows initial viral RNA and DNA values for subjects (8, 9, 12, and 15) and subsequent values for each subject on the same line (at respective times 166, 191, 284 and 47 d from treatment initiation), obtained at the time that a change in the polymerase coding capacity was detected (see Supplementary Table S1). For two additional subjects (13 and 14), viral RNA values are available at the initial and subsequent time points, but no changes in polymerase coding capacity were detected. The logarithmic mean, 95% confidence intervals, and results of a two-tailed paired sample t test are presented for comparisons of the values at these time points. All of the viral RNA and DNA values and polymerase changes are provided in Supplementary Table S1.

The portion of the *pol* gene encoding residues 41-219 of reverse transcriptase was sequenced from PBMCs obtained at the same time points used for viral RNA measurements. Changes in coding sequences were found at 0-5 residues over the course of therapy in different patients (Supplementary Table S1). Changes in *pol* were found in 4 of 6 patients with increasing viral RNA levels (subjects 8, 9, 12, and 15) and 2 of 4 patients that did not manifest increasing viral RNA levels (subjects 10 and 16 versus subjects 3 and 11). In the subjects with changes in *pol* and increasing viral RNA levels, these changes occurred at the same time (Fig 2 and Supplementary Table S1). Overall, eight of twelve patients had changes in *pol* sequence and/or increases in viral RNA expression, during the course of therapy, and a ninth patient had variable high levels of viral RNA throughout the treatment course.

### Responses

**Figure 3:**
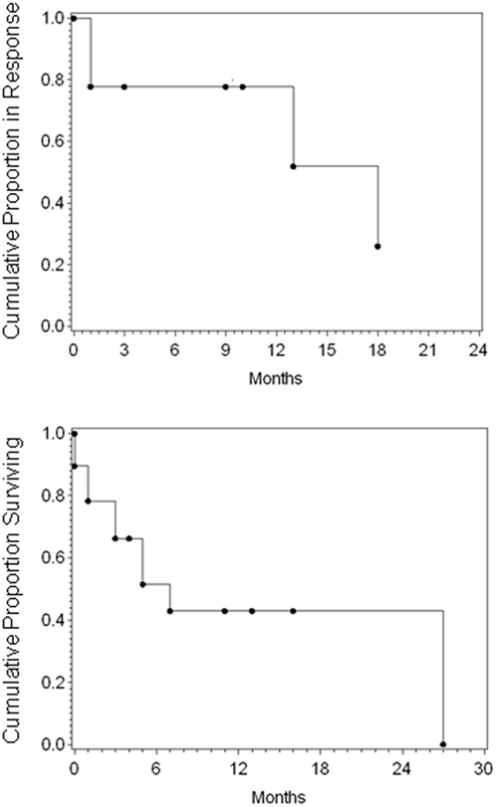
Duration of response and disease-free survival of all patients on the study.

Eleven of twelve evaluable patients (or 11 of 19 total patients) had responsive disease, including two complete remissions and nine partial remissions, for an overall response rate of 91% for evaluable patients and 58% for all patients (95% confidence intervals from 60% to 99% and 36% to 77%, respectively). Disease relapsed in four patients after 1, 1, 13, and 18 mos, respectively. Progression-free and overall survival curves are shown in Fig 3. Nine patients are alive at the time of this report, at 0, 1, 3, 4, 5, 7, 11, and 16 mos after completion of therapy.

## Discussion

The response rates, duration of responses, and overall survival of the current trial are similar to previous trials of ATLL. The most recent published Japanese cooperative ATLL trial utilized the LSG15 protocol, and included 58 patients with leukemic ATLL, 28 patients with lymphoma type, and 10 patients with unfavorable chronic ATLL [24]. Patients received seven cycles of VCAP (vincristine, cyclophosphamide, doxorubicin, and prednisone), AMP (doxorubicin, ranimustine, and prednisone), and VECP (vindesine, etoposide, carboplatin, and prednisone). This resulted in 35% complete remissions and 45% partial remissions, median survival of 13 mos, and 31% 2-yr survival.

The limitations of the current trial include the small number of patients, and several patients were non-evaluable. The high proportion of non-evaluable patients is likely due to the intensity of the treatment regimen and the poor prognostic features of the enrolled patients. In addition, only six patients received antivirals, and no patient received the planned 12 mos of antiviral therapy, making it impossible to evaluate the relative contribution of this therapy in the trial.

Viral DNA measurements were feasible, but did not have prognostic or predictive power. However, results of viral RNA measurements were quite unexpected. Although much of the literature suggests that little if any viral RNA is expressed in patients at the time of presentation with ATLL [25]–[28], in fact, the majority of our patients had detectable *tax* viral RNA at diagnosis, including 3 of 15 patients with high levels of viral RNA at presentation. During the course of therapy, viral RNA levels were measured on subsequent samples in 12 subjects. In 6 of these subjects, increased viral RNA levels were found on subsequent measurements, and in one other patient with a high initial viral level, variably high levels were found at subsequent measurements. In 4 of 6 patients with increasing viral RNA levels during the course of therapy, changes were first identified in the reverse transcriptase open reading frame at the same time. Sequence changes in reverse transcriptase did not correspond with those predicted to give rise to zidovudine or lamivudine-resistance based on studies of HIV-1 reverse transcriptase, and generally preceeded antiviral therapy [29]. These sequence changes may have arisen from selection of rare viral variants, or more likely, as a result of error-prone retrovirus replication. Such sequence changes have not been described in patients who are not undergoing therapy [30]–[32].

The finding of virus expression and mutation raises questions as to whether or not these activities are related to ATLL relapse or progression. If there is such a relationship, virus expression and mutation could be a marker of progression, perhaps a manifestation of lymphocyte activation [33]. Alternatively, virus expression and mutation could be a cause of progression, possibly through generation of clonal heterogeneity of infected tumor cells. This could result from changes in the viral integration site which could select for expression or inactivation of critical cellular sequences, or for integration in sites that are more transcriptionally active [25]. Alternatively, this could be due to therapy-mediated clearance of clonal ATLL cells carrying quiescent virus, and subsequent repopulation and infection of T cells with HTLV-1. Changes in integration sites during the treatment course were confirmed in several of these patients (not shown). Another possibility is that virus expression or mutation results in secondary genetic events that select for tumor progression, independent of new integration events [34]. In either event, incorporation of antiviral therapy earlier in the course of therapy could be beneficial.

Although most evaluable patients achieved responses, responses were not long, with relapses occurring during or shortly after completion of chemotherapy in many patients. Thus, more effective therapies are required. Although the leukemic form of ATLL responds well to zidovudine and alpha interferon-2a, the lymphomatous form is poorly responsive to most therapies [16], [17]. Various strategies might be considered to improve current chemotherapy regimens, such as simultaneous use of chemotherapy and antiviral agents, addition of a proteasome inhibitor or other agent to overcome NFκB-induced chemotherapy resistance [35], [36], or p53 inactivation in tumor cells [37]. Alternatively, chemotherapy combined with antibody or antibody conjugates may be considered [38]. Approaches using allogeneic stem cell transplantation, perhaps earlier in the course of disease, may be appropriate for younger patients for whom a donor can be identified [39]. The current trial of chemoantiviral therapy and the translational studies of viral RNA provide novel insights to improve therapy for this hematopoietic malignancy.

## Supporting Information

Table S1 (0.10 MB DOC)

Checklist S1 CONSORT Checklist
(0.06 MB DOC)

Protocol S1 Trial Protocol
(0.32 MB PDF)
