# Supplementary material for: Human T Cell Leukemia Virus Reactivation with Progression of Adult T-Cell Leukemia-Lymphoma
Source: PLoS One. 2009 Feb 10;4(2):e4420. doi: 10.1371/journal.pone.0004420 (PMC2636875; doi:10.1371/journal.pone.0004420)
Supplement: Table S1 — (0.10 MB DOC) [file pone.0004420.s001.doc]

| **Table S1. Virological Data from Subjects on Study** | | | | | |
| --- | --- | --- | --- | --- | --- |
| Subject # | Days | Cycle | DNA | RNA | Pol Sequence |
|  |  |  | (x100) | (x10,000) |  |
| 1 | 0 | 1 | 36 | 0 | K65M, Y166H |
| 2 | 0 | 1 | 2 | 0 | nd |
| 3 | 0 | 1 | 163 | 0 | Y166H, H194R |
|  | 27 | off | 150 | 0 | Y166H, H194R |
| 4 | 0 | 1 | 38 | 180 | A174V |
| 5 | 17 | off | 41.5 | 0.2 | nd |
| 6 | 0 | 1 | nd | 16 | Y166H |
| 7 | 0 | 1 | 106 | 283000 | Y166H |
| 8 | 0 | 1 | 31 | 24 | Q123R, Y166H |
|  | 166 | off | 189 | 3340 | Q123R, V159F, Y166H |
| 9 | 0 | 1 | 31 | 0 | nd |
|  | 44 | 3 | 48 | 0 | nc |
|  | 94 | 5 | 0 | 0 | A174V |
|  | 153 | 6 | 49 | 0 | nd |
|  | 191 | 7 | 61 | 240 | E45G, P51L, Y52N, F61T, A174V |
| 10 | 43 | 3 | nd | nd | R77G, Y166H |
|  | 99 | 5 | 41 | 0 | R77G, Y166H |
|  | 169 | 50 | 10 | 0 | R77G, L86F, Y166H, S210F |
| 11 | 0 | 1 | 4 | nd | nd |
|  | 46 | 3 | 24 | 0 | Y166H |
|  | 88 | 50 | nd | 0 | Y166H |
| 12 | 0 | 1 | 0 | 0 | Y166H |
|  | 249 | 53 | 0 | 10000 | Y166H |
|  | 284 | 54 | 8 | 48900 | V62F, K65V, Y166H |
| 13 | 0 | 1 | 144 | 4 | N137H, Y166H |
|  | 42 | 3 | 6 | 47 | N137H, Y166H |
| 14 | 0 | 1 | 81 | 7 | Y166H |
|  | 85 | 3 | 4 | 4900 | Y166H |
|  | 124 | 5 | 19 | 44100 | Y166H |
| 15 | 0 | 1 | 10 | 8.7 | Q123G, Y166H |
|  | 47 | 3 | 8.3 | 186 | Q123G, F130S |
| 16 | 48 | 3 | 16 | 4200 | Y166H |
|  | 139 | 50 | 31 | 1.4 | Y166H, L214S |
|  | 230 | off | 12 | 0.4 | Y166H, D196G |
| 17 | 0 | 1 | 22 | 14000 | nd |
|  | 85 | 5 | 9.5 | 2250 | nd |
|  | 117 | 51 | 97 | 5660 | nd |
| 18 | 0 | 1 | 2 | 0 | Y166H |
| 19 | 28 | 2 | 13 | 1.6 | nd |
|  | 55 | 3 | 12 | 0.6 | K147R, Y166H, L198Q |
|  | 171 | 50 | 14 | 55 | K147R, Y166H, L198Q |
|  | 250 | 53 | nd | nd | K147R, Y166H, L198Q |
|  | 348 | 56 | 19 | 1.8 | K147R, Y166H, L198Q |
|  |  |  |  |  |  |
| Abbreviations: | | Values for odd number subjects in shaded boxes | | | |
|  |  | Days=Days from treatment initiation | | | |
|  |  | Cycle=Cycle of chemo initiated on that day for 1-7 or | | | |
|  |  | 50 for initiation of antivirals, or 51-56 for 1-6 mos of antivirals | | | |
|  |  | off - indicates patient has come off protocol | | | |
|  |  | RNA=viral pX RNA/10,000 copies hprt RNA; | | | |
|  |  | RNA limit of detection of 0.5 | | | |
|  |  | DNA=viral DNA/100 PBMCs | | | |
|  |  | Pol sequences are compared to HTLV-1 strain ACH | | | |
|  |  | nc; not changed from ACH sequence; nd, not done | | | |
|  |  | Boxed values obtained during antiviral therapy | | | |
