# Supplementary material for: Human T Cell Leukemia Virus Reactivation with Progression of Adult T-Cell Leukemia-Lymphoma
Source: PLoS One. 2009 Feb 10;4(2):e4420. doi: 10.1371/journal.pone.0004420 (PMC2636875; doi:10.1371/journal.pone.0004420)
Supplement: Protocol S1 — Trial Protocol (0.32 MB PDF) [file pone.0004420.s003.pdf]

# AMC
# AIDS ASSOCIATED MALIGNANCIES CLINICAL TRIAL CONSORTIUM

## AMC #033

## PHASE II TRIAL OF INDUCTION THERAPY WITH EPOCH CHEMOTHERAPY AND MAINTENANCE THERAPY WITH COMBIVIR/INTERFERON ALPHA-2a FOR HTLV-1 ASSOCIATED T-CELL NON-HODGKIN'S LYMPHOMA

A Multicenter Trial of the AIDS Malignancy Consortium

Sponsored by:
The National Cancer Institute
Division of Cancer Treatment and Diagnosis

**Protocol Chairs**
Lee Ratner, MD, PhD

**Protocol Co-Chairs**
William Harrington, MD
Alexandra Levine, MD

**AMC #033 Version 8.0 06/03/05**
***NCI Version Date 06/03/05***

# PROTOCOL TEAM ROSTER

## AMC #033
## PHASE II TRIAL OF INDUCTION THERAPY WITH EPOCH CHEMOTHERAPY AND MAINTENANCE THERAPY WITH COMBIVIR/INTERFERON ALPHA-2a FOR HTLV-1 ASSOCIATED T-CELL NON-HODGKIN'S LYMPHOMA


**Protocol Chair:**
Lee Ratner, MD, PhD
Washington University School of Medicine
Division of Oncology
Box 8069
660 S Euclid Ave
Washington University
St Louis, MO 63110
Telephone: 314-362-8836
Fax: 314-747-2120
Email: LRATNER@IMGATE.WUSTL.EDU

**Protocol Co-Chairs:**
William J. Harrington, Jr., MD
Associate Professor of Medicine
University of Miami School of Medicine
Sylvester Comprehensive Cancer Center
1475 N.W. 12th Avenue, Ste. 3400 (D8-4)
Miami, Florida 33136
Phone: 305-243-4994
Fax: 305-243-5239
Email: wharring@med.miami.edu

Alexandra M. Levine, MD
USC School of Medicine
Norris Cancer Hospital
1441 Eastlake Ave., MS-34, Rm 3468
Los Angeles, California 90089
Phone: 323-865-3913
Fax: 323-865-0060
Email: hornor@hsc.usc.edu

**Protocol Statistician:**
Jeannette Lee, Ph.D.
Research Professor of Medicine
AMC Operations and Statistical Center
2001 Third Avenue South , Rm # 1078
Birmingham, Alabama 35233
Phone: 205-975-5165
Fax: 205-975-7453
Email: jylee@uab.edu

**Project Coordinator:**
Karen Cole, B.S.
AMC Operations and Statistical Center
University of Alabama at Birmingham
2001 Third Avenue South, Rm # 1078
Birmingham, Alabama 35233
Phone: 205-934-5165
Fax: 205-975-7453
Email: karen.cole@ccc.uab.edu

**Protocol Liaison**:
Lisa Cabral, RN
University of Miami School of Medicine
1475 NW 12th Ave, Suite 3400 (D8-4)
Miami, FL 33136
Phone: 305-243-4994
Fax: 305-243-5239
Email: lcabral@med.miami.edu

**AMC #033**
**This protocol is open to enrollment at all AMC sites.**

## Table of Contents


1.0 INTRODUCTION ........ 5
1.1 HTLV-1 INFECTION ........ 5
1.2 HTLV-1 ASSOCIATED NON-HODGKIN'S LYMPHOMA ........ 6
1.3 HTLV-1 PROVIRAL DNA AND CELL-ASSOCIATED AND VIRION RNA LOAD AND PROTEIN EXPRESSION ........ 6
1.4 HTLV-1 CLONALITY ........ 7
1.5 APOPTOSIS MARKERS IN HTLV-1 INFECTED CELLS ........ 7
1.6 TREATMENT OF HTLV-1 ASSOCIATED NON-HODGKIN'S LYMPHOMA ........ 7
1.7 ANTIVIRAL THERAPY FOR HTLV-1 INFECTION ........ 8
1.8 EPOCH CHEMOTHERAPY ........ 8
1.9 RATIONALE ........ 8
2.0 OBJECTIVES ........ 9
2.1 PRIMARY ENDPOINT ........ 9
2.2 SECONDARY ENDPOINTS ........ 9
3.0 SCHEMA ........ 9
3.1 TITLE ........ 9
3.2 POPULATION ........ 9
3.3 TREATMENT ........ 9
3.4 ENDPOINTS ........ 10
4.0 PATIENT SELECTION ........ 10
4.1 INCLUSION CRITERIA ........ 10
4.2 EXCLUSION CRITERIA ........ 11
4.3 ENROLLMENT PROCEDURES ........ 11
5.0 REQUIRED DATA ........ 12
5.1 TESTS AND OBSERVATIONS ........ 12
5.2 RECORDS TO BE KEPT ........ 12
5.3 ROLE OF DATA MANAGEMENT ........ 13
6.0 STUDY MEDICATIONS ........ 13
6.1 CHEMOTHERAPY MEDICATIONS ........ 13
6.2 ANTIRETROVIRAL AGENTS ........ 15
6.3 DRUG SUPPLY ........ 15
6.4 CHEMOTHERAPY ADMINISTRATION ........ 15
6.5 ANTIRETROVIRAL (ARV) ADMINISTRATION ........ 16
6.6 CONCURRENT MEDICATION ........ 16
6.7 CNS PROPHYLAXIS ........ 17
6.8 MENINGEAL LYMPHOMA ........ 17
6.9 RADIOTHERAPY ........ 17
7.0 CLINICAL AND LABORATORY EVALUATIONS ........ 18
7.1 BASELINE/PRETREATMENT EVALUATION ........ 18

7.2 EVALUATIONS DURING CHEMOTHERAPY TREATMENT ........ 19
7.3 EVALUATIONS DURING ANTIRETROVIRAL (ARV) TREATMENT ........ 20
7.4 POST-TREATMENT EVALUATION ........ 20
7.5 EARLY DISCONTINUATION OF THERAPY ........ 21

8.0 DOSE MODIFICATION/TOXICITY MANAGEMENT ........ 21
8.1 CHEMOTHERAPY MODIFICATIONS: HEMATOLOGIC TOXICITY ........ 21
8.2 CHEMOTHERAPY MODIFICATIONS: NON-HEMATOLOGIC TOXICITY ........ 22
8.3 ARV TOXICITY MANAGEMENT ........ 22

9.0 CRITERIA FOR TREATMENT DISCONTINUATION ........ 24
9.1 PATIENT WITHDRAWAL ........ 24

10.0 REPORTING OF ADVERSE EVENTS ........ 24
10.1 CLASSIFICATION OF ADVERSE EVENTS BY SEVERITY AND RELATIONSHIP TO STUDY DRUG ........ 24
10.2 ADVERSE EVENTS WITH COMMERCIAL AGENTS ........ 25

11.0 EVALUATION OF RESPONSE ........ 26
11.2 DEFINITION OF RESPONSE ........ 27

12.0 STATISTICAL CONSIDERATIONS ........ 30
12.1 SAMPLE SIZE ESTIMATION AND ACCRUAL ........ 30
12.2 STATISTICAL ANALYSIS PLAN ........ 30
12.3 DATA SAFETY MONITORING ........ 30

13.0 ETHICAL AND REGULATORY CONSIDERATIONS ........ 31
13.1 INFORMED CONSENT ........ 31
13.2 WOMEN AND MINORITIES ........ 31

14.0 REFERENCES ........ 32

Appendix I: Karnofsky Performance Scale ........ 35

Appendix II: Ann Arbor Staging Criteria ........ 36

Appendix III: Central Pathology Review ........ 37

Appendix IV: Schedule Of Evaluations ........ 38

Appendix V: Blood For HTLV-1 Proviral Load, Clonality, RNA & Protein Expression, Plasma Virus Load, & Apoptosis Markers ........ 39

Appendix VI: Informed Consent ........ 41

Appendix VII: Grid of Forms Submission Table ........ 49

Appendix VIII: ACSB Informed Consent ........ 50

Appendix IX: ACSB Specimen Preparation & Shipping Instructions ........ 53

Appendix X: Data Safety And Monitoring Plan ........ 55

# 1.0 INTRODUCTION

## 1.1 HTLV-1 Infection

Human T-cell leukemia virus type 1 (HTLV-1) is a member of oncornaretrovirus family[1]. It is closely related to simian T-cell leukemia virus type 1 (STLV-1), and phylogenetic trees show that STLV-1 and HTLV-1 strains are intermingled [2]. A somewhat more distantly related virus, with 60% nucleotide sequence homology to HTLV-1, is HTLV-2. FDA approved serological assays for HTLV do not reliably distinguish HTLV-1 and HTLV-2. However, these infections can be distinguished by antibody reactivity with recombinant proteins or by polymerase chain reaction assays.

HTLV-1 infections are prevalent in southern Japan, the Caribbean, and many parts of Central and South America, the Middle East, and Africa, where 10-15% of the population is infected [1,3]. In the United States, 0.25% of volunteer blood donors are infected with HTLV-1 or HTLV-2. Infections by both viruses are not unusual among intravenous drug abusers. HTLV-2 is endemic in native North, Central, and South American populations.

The HTLV-1 genome includes *gag, pro, pol,* and *env genes* encoding the inner capsid of the virion, the viral protease, the viral reverse transcriptase and integrase enzymes, and the glycoprotein on the virion surface necessary for viral entry [4]. Accessory genes include *tax*, encoding a potent transcriptional trans-activator, and *rex*, which encodes a mediator of nuclear export of viral RNAs. Other accessory genes encode proteins designated p12, p13, p27, and p30, whose functions remain to be fully characterized.

There is considerable evidence that the *tax* gene product, Tax, is critically important for the leukemogenic activity of the virus [5, 6]. Although most viral genes are not expressed in ATLL samples, Tax expression is usually detectable. Tax together with ras is capable of transforming rodent fibroblasts, and when expressed in a retrovirus or Herpes *samirii* vector is capable of immortalizing human lymphocytes. Constitutive Tax expression in transgenic mice results in several different types of malignancies, whereas expression in the lymphoid compartment of a transgenic mouse results in a lymphoproliferative malignancy. Tax functions at transcriptional and post-transcriptional steps. Tax up-regulates several transcriptional pathways to enhance the expression of the viral promoter as well as several interleukins and their receptors (e.g. IL2, IL2Rα, IL15), cytokines (e.g.

IFNγ, GM-CSF), adhesion molecules (e.g. ICAM-1, VCAM-1, VLA-4), growth promoting factors (e.g. c-myc, c-sis, c-fos, egr-1, cyclin D2, and E2F-1), and apoptosis inhibiting factors (e.g. bcl xL, A20). Moreover, Tax can directly bind and enhance or inhibit the activity of several growth promoting (e.g. cyclins D2 and D3, E2F) or inhibiting factors (e.g. p15, p16, and p53 tumor suppressor proteins and cell cycle checkpoint protein MAD1), respectively. In addition Tax promotes the phosphorylation and inactivation of the p53 tumor suppressor protein.

## 1.2 HTLV-1 Associated non-Hodgkin's Lymphoma

Although HTLV-2 is not clearly associated with a clinical disorder, HTLV-1 is closely associated with two clinical disorders [(1)]. About 5% of HTLV-1 infected individuals eventually develop a form of myelopathy known as HTLV-1 myelopathy (HAM) or tropical spastic paraparesis (TSP). It is characterized by lower limb spasticity, bowel and bladder disturbances, and slow but steady progression over several years. Additionally, about 5% of HTLV-1 infected individuals develop a T-cell non-Hodgkin's lymphoma designated adult T-cell leukemia lymphoma (ATLL). It occurs almost exclusively in individuals who acquired HTLV-1 as a result of breast feeding, although four or five decades are generally required before development of disease. ATLL is characterized by frequent blood and bone marrow involvement, hypercalcemia, and lytic bone lesions. It is most commonly a CD4+ leukemia/lymphoma, although occasional examples of CD8+ lymphoma have been described. ATLL may fit into a variety of pathological subtypes as classified by the International Working Party Formulation. Clinical classifications of ATLL have included a "smoldering" disorder characterized by rash and minimal blood involvement, as well as a chronic form of ATLL, which have median survivals of two years or more [(7)]. In contrast, a "lymphomatous" form of ATLL, and an "acute" form of ATLL have median survivals of 3-6 mos.

## 1.3 HTLV-1 Proviral DNA and Cell-Associated and Virion RNA Load and Protein Expression

Recently assays of HTLV-1 proviral DNA load using PCR techniques have been developed and applied to clinical samples. These studies demonstrated levels of about

0.02 copies/PBMC in asymptomatic individuals and 0.08 copies/PBMC in HAM patients[8].

RT-PCR assays of cell-associated viral and virion RNA have been developed and used to demonstrate that the majority of infected cells do not express viral products [9]. However, quantitative data on transcript levels has not been obtained with clinical samples.

### 1.4 HTLV-1 Clonality

In ATLL, there is a clonal expansion of T cells with clonal T-cell receptor gene rearrangements and clonal sites of provirus integration into chromosomal DNA, as determined by Southern blot hybridization [10]. More recently, a ligation mediated PCR technique has been utilized as a more sensitive way to detect different clonal populations in asymptomatic infected individuals, HAM patients, and ATLL patients [11]. These studies have demonstrated that the expanded clone of malignant ATLL has arisen from an oligoclonal expansion of HTLV-1 infected cells. These studies suggest that HTLV-1 dissemination can occur by viral replication and clonal expansion of infected cells.

### 1.5 Apoptosis Markers in HTLV-1 Infected Cells

HTLV-1 infected cells are resistant to inducers of apoptosis. This may be a result of Tax inactivation of the p53 gene, or up-regulation of apoptosis inhibitor A20 or bcl-XL [5, 6]. Arsenic trioxide and Interferon Alpha-2a have been used to induce apoptosis in HTLV-1 infected T-cell lines [12].

### 1.6 Treatment of HTLV-1 Associated non-Hodgkin's Lymphoma

Various chemotherapy regimens have been utilized to treat patients with ATLL with complete response rates of only about 30%, which are not durable [13-16]. In a large cooperative study in Japan, 4-year survival of patients with acute or lymphomatous forms of ATLL was only 4%.

Two reports describe major response rates of 58% and 100%, respectively, in the lymphomatous form of ATLL, with the use of a combination of azidothymidine and Interferon Alpha-2a [17, 18]. However, other investigators reported lower response rates with this approach [19].

Other approaches have included the use of antibodies to IL2Rα with or without conjugates to radioisotopes, or the use of an IL2-diptheria toxin product [20].

## 1.7 Antiviral Therapy for HTLV-1 Infection

Although the AZT/Interferon Alpha-2a combination was used in ATLL patients, it is unclear whether this combination had antiviral, antitumor, and/or immunomodulatory activity towards HTLV-1. A recent report of the use of lamivudine in 5 HAM patients suggested antiviral activity, as demonstrated by a 1 log median decrease in virus load [21]. This was associated with clinical improvement in one patient. Use of anti-retroviral agents has recently been shown to decrease HTLV-1 proviral DNA.

## 1.8 EPOCH Chemotherapy

The current trial will utilize EPOCH chemotherapy, which provides a 5-day continuous infusion of etoposide, vincristine, and doxorubicin, in addition to an intravenous bolus of cyclophosphamide and six days of oral prednisone. The selection of infusional chemotherapy is based on the poor response durations of bolus chemotherapy (see section 1.2), and since EPOCH chemotherapy has produced complete remissions in 25-50% of aggressive newly diagnosed or refractory non-Hodgkin's lymphomas in the non-HIV patient population [22, 23]. Moreover EPOCH chemotherapy used in aggressive HIV-associated non-Hodgkin's lymphomas has produced a complete remission rate of 78%, and was well tolerated [24]. Our anecdotal experience of EPOCH chemotherapy in two patients with ATLL has demonstrated efficacy and tolerability (W.H., unpublished). Moreover, EPOCH chemotherapy is feasible in areas of the world in which HTLV-1 infection is endemic.

## 1.9 Rationale

The rationale of the current study is to explore the use of combination chemotherapy together with antiretroviral agents in order to determine the efficacy and toxicity of this approach, while also examining markers of virus replication and expression, and tumor cell proliferation to gain understanding of the biological basis of this malignancy and to identify predictors of response.

## 2.0 OBJECTIVES

### 2.1 Primary endpoint

To determine the efficacy (response rate) of EPOCH chemotherapy followed by antiretroviral therapy in patients with HTLV-1 associated leukemia/lymphoma (ATLL).

### 2.2 Secondary endpoints

1. To determine the duration of response of EPOCH chemotherapy followed by antiretroviral therapy for HTLV-1 associated leukemia/lymphoma (ATLL).
2. To evaluate the effects of EPOCH chemotherapy followed by antiretroviral therapy on HTLV-1 DNA and RNA load. To determine if relapsed or progressive disease is a result of renewed virus replication.
3. To evaluate the effects of EPOCH chemotherapy followed by antiretroviral therapy on HTLV-1 DNA clonality in order to determine if relapsed or progressive disease is a result of expansion of the original predominant infected T cell clone, and to evaluate the effects of EPOCH chemotherapy followed by antiviral therapy on Tax protein expression, p53 function, markers of apoptosis, and the development of 3TC-resistant virus.

## 3.0 SCHEMA

### 3.1 Title

Phase II Trial of Induction Therapy with EPOCH Chemotherapy and Maintenance Therapy with Combivir/Interferon Alpha-2a for HTLV-1 Associated Adult T Cell Leukemia/Lymphoma.

### 3.2 Population

32 patients with HTLV-1 associated ATLL.

### 3.3 Treatment

EPOCH chemotherapy will be administered as follows:

Etoposide 50 mg/m$^2$/day given as a continuous 96 hr IV infusion on days 1-5.

Vincristine 0.4 mg/m$^2$/day given as a continuous 96 hrs IV infusion on days 1-5.

Doxorubicin 10 mg/m$^2$/day given as a continuous 96 hrs IV infusion on days 1-5.

Cyclophosphamide 750 mg/m$^2$ given IV on day 5. The infusion time for cyclophosphamide is 30 minutes.

Prednisone 60mg/m$^2$ given orally on days 1-5.

Cycles will be repeated every 21-28 days, for two cycles beyond best response, and a maximum of 6 cycles. "Best response" is the response achieved when 1 or more additional cycles of chemotherapy are given and no additional tumor shrinkage is noted. That may include stable or progressive disease after 2 cycles chemotherapy.

**Plus** G-CSF: All patients will receive G-CSF at a dose of 5ug/kg subcutaneously daily beginning 24 hours after the administration of prednisone for 10 days beginning on day 6 or until the absolute neutrophil count has recovered to>4,000 cell/mm$^3$

**Plus** Antiviral therapy for one year will begin one month after completion of EPOCH or will start earlier if the investigator feels that it is medically indicated, and will consist of:

Combivir (zidovudine 300 mg + lamivudine 150 mg) 1 tablet po bid.

Interferon Alpha-2a 9 mU SQ qd.

### 3.4 Endpoints

1. Response rate
2. Response duration
3. Relapse rate
4. Toxicity
5. Effect of treatment on HTLV-1 DNA and RNA expression, Tax protein expression, DNA clonality, p53 function, apoptosis markers, and 3TC/ZDV-resistance.

## 4.0 PATIENT SELECTION

### 4.1 Inclusion Criteria

4.1.1 Histologically or cytologically documented ATLL. Patients with previously treated ATTL are eligible.

4.1.2 Tumors must be CD3 positive (>50% cells express CD3).

4.1.3 Documented HTLV-1 infection: documentation may be serologic assay (ELISA, Western blot) and confirmed to be HTLV-1 rather than HTLV-2 by differential Western blot (e.g., Genelabs Diagnostics HTLV Blot 2.4) or PCR.

4.1.4 Evaluable or measurable disease.

4.1.5 All stages are eligible.

4.1.6 Adequate hematologic function: ANC>1000 cells/mm$^3$, platelet count>75,000/mm$^3$ unless cytopenias are secondary to ATLL. All patients must

be off hematologic growth factors for at least 24 hours prior to initiation of chemotherapy.

4.1.7 Adequate hepatic function: transaminase<7 times the upper limit of normal, biliribuin<2.0, unless secondary to hepatic infiltration with lymphoma or isolated in-directed hyperbilirubinemia associated with the use of indinavir. For bilirubin>3.0 due to hepatic involvement, vincristine and doxorubicin will not be given in cycle 1.

4.1.8 Creatinine<2.0 unless due to lymphoma.

4.1.9 KPS $\geq$ 50.

4.1.10 Age $\geq$ 18 years.

4.1.11 Able to give informed consent

4.1.12 Female patients must have a negative pregnancy test within 72 hours of entering into the study. Males and females must agree to use two methods of birth control during the study and for 6 months after the discontinuation of Interferon Alpha-2a. Women must avoid pregnancy and men avoid fathering children while in the study.

4.1.13 HIV positive patients are eligible.

## 4.2 Exclusion Criteria

4.2.1 Acute active opportunistic infection requiring acute therapy. Chronic therapy with potentially myelosuppressive agents is allowed provided that entry hematologic criteria are met.

4.2.2 Concurrent malignancy excluding in situ cervical cancer, or non-metastatic non-melanomatous skin cancer.

4.2.3 Patients with untreated thyroid disease, autoimmune disease, uncontrolled significant psychiatric disease, and women who are breastfeeding have been added to the protocol. Women who are breastfeeding are excluded because the chemotherapy agents may be found in breast milk.

## 4.3 Enrollment Procedures

This study will be available for enrollment at all AMC sites. Participating sites must have this protocol approved by their Institutional Review Boards (IRB) and be registered with the AMC Operations Center.

After eligibility has been determined and an informed consent has been signed by the patient, they must be registered. Registration will be accepted from the AMC main or affiliated institutions. Registration must occur prior to initiation of the baseline (Day 1) visit. You may call the AMC Project Coordinator at (205) 934-5165, Monday through Friday, 8 AM to 5 PM, Central Standard Time. Fax the following information to (205) 975-7453:

1. Your name, telephone and FAX numbers
2. AMC protocol and version number
3. Your principal investigator's name and your institution site number
4. Patients race, sex, date of birth and method of insurance payment
5. Three letters for patient's initials
6. Date the informed consent was signed
7. Date of enrollment
8. Completed Eligibility Form for Protocol

The AMC Project Coordinator will assign a study identification number to the patient. This study identification number is to be used on all data collection forms for the protocol. Confirmation of registration will be sent to the AMC institution.

## 5.0 REQUIRED DATA

### 5.1 Tests and observations

See section 7.0. Unless otherwise noted, all tests must be completed within 30 days of study entry.

### 5.2 Records to be Kept

Case Report Forms (CRFs) will be provided for each subject. Subjects must not be identified by name on any study documents. Subjects will be identified by a Patient Identification Number upon registration.

All data on the CRF must be legibly recorded in black ink or typed. A correction should be made by striking through the incorrect entry with a single line and entering the correct information adjacent to it. The correction must be initialed and dated by the investigator

or designated qualified individual. Any requested information that is not obtained as specified in the protocol should have an explanation noted on the CRF as to why the required information was not obtained.

## 5.3 Role of Data Management

5.3.1 Instructions concerning the recording of study data on CRFs will be provided by the Operations Center.

5.3.2 It is the responsibility of the AMC Operations Center to assure the quality of data for their study. This role extends from protocol development to generation of the final study database.

## 6.0 STUDY MEDICATIONS

## 6.1 Chemotherapy Medications

**ETOPOSIDE:** Etoposide or VP-16 is an epipodophyllotoxin derived from the mandrake plant Podophyllum peltatum. It is a cell cycle phase specific agent that blocks topoisomerase II. It has a biphasic half-life and is eliminated by both renal clearance and metabolism.
The major and dose-limiting toxicity of etoposide is myelosuppression. Constipation, diarrhea, dysphagia, aftertaste, abdominal pain, stomatitis, and anorexia have also been reported. Mucositis and hepatotoxicity are seen primarily with high doses. Transient hypotension and other anaphylactic-like symptoms are associated with rapid infusion. Please refer to the approved package insert for complete prescribing and toxicity information.

**VINCRISTINE SULFATE**: Vincristine sulfate is a vinca alkaloid from the plant Cantharanthus roseus. It acts by binding to or crystallizing microtubular proteins of the mitotic spindle. It is a cell cycle phase specific agent and can also affect DNA directed RNA polymerase. It has a triphasic half-life and primary elimination is by the liver into the bile and feces. The major and dose-limiting side effect of vincristine is neurotoxicity. The main manifestation is a mixed sensorimotor peripheral neuropathy. Reduced or loss of deep tendon reflexes, paresthesias, weakness, myalgias, and motor disturbances may occur. Autonomic toxicity also occurs which may cause constipation, obstipation,

abdominal cramps, and ileus. It has mild myelosuppressive effects and is a vesicant causing local necrosis at the site of injection if extravasation occurs. Please refer to the approved package insert for complete prescribing and toxicity information.

**DOXORUBICIN:** Doxorubicin is an anthracycline antibiotic which binds tightly with DNA, inhibits nucleic acid synthesis and causes DNA strand breaks. Although active throughout the cell cycle, cells in S phase are most sensitive.
Common side effects include myelosuppression, alopecia, and stomatitis which is dose related and may be severe. Drug-induced cardiomyopathy which may result in congestive heart failure is a cumulative dose-dependent effect and risk becomes considerable at total doses exceeding 500 mg/m$^2$. Doxorubicin is given intravenously and is a vesicant causing severe local necrosis at the site of injection if extravasation occurs. Nausea and vomiting are frequent. Please refer to the approved package insert for complete prescribing and toxicity information.

**CYCLOPHOSPHAMIDE**: Cyclophosphamide is an alkylating agent and is cell cycle nonspecific. It causes cross linking of DNA and is the most active single agent in the treatment of non-Hodgkin's lymphoma. Side effects of cyclophosphamide include nausea, vomiting, myelosuppression, and alopecia. Sterility and testicular atrophy are common in men and amenorrhea is seen in women. Hemorrhagic cystitis is caused by metabolites of cyclophosphamide excreted through the urine. Bladder irritation can be reduced by adequate hydration. Please refer to the approved package insert for complete prescribing and toxicity information.

**PREDNISONE**: Prednisone is a corticosteroid and its mechanism of action as a cytotoxic agent is not clearly understood. Short term use produces minimal side effects but prolonged use is associated with hypertension, hyperglycemia, myopathy, osteoporosis, pancreatitis, and immunosuppression. Alterations in mood and insomnia are common acute side effects. Please refer to the approved package insert for complete prescribing and toxicity information.

## 6.2 Antiretroviral Agents

**COMBIVIR**: Combivir is a fixed combination of 300 mg zidovudine (AZT) and 150 mg of lamivudine (3TC). Both agents are nucleoside analog inhibitors of HIV-1 reverse transcriptase. Side effects of zidovudine include headache, fever, rash, nausea, anorexia, diarrhea, pain, vomiting, anemia, leukopenia, granulocytopenia, and weakness. Less commonly, malaise, dizziness, insomnia, somnolence, hyperpigmentation of nails, dyspepsia, and paresthesias occur. Rarely, neurotoxicity, confusion, mania, seizures, hepatotoxicity, cholestatic jaundice, or myopathy occur. Side effects of lamivudine include headache, insomnia, malaise, fatigue, pain, nausea, diarrhea, vomiting, peripheral neuropathy, paresthesias, nasal symptoms, and cough. Rarely, dizziness, depression, fever, chills, rash, anorexia, abdominal pain, dyspepsia, elevated amylase, neutropenia, anemia, elevated AST, myalgias, or arthralgias occur. Please refer to the approved package insert for complete prescribing and toxicity information.

**INTERFERON ALPHA-2**a: Interferon Alpha-2a is an immune modulator, with antiviral and antitumor activity. Side effects of Interferon Alpha-2a include dizziness, fatigue, malaise, fever, chills, rash, xerostomia, nausea, vomiting, diarrhea, abdominal cramps, weight loss, metallic taste, myelosuppression, leukopenia, anemia, thrombocytopenia, anemia, diaphoresis. Rarely, headache, delirium, somnolence, neurotoxicity, alopecia, dry skin, anorexia, stomatitis, hepatotoxicity, peripheral neuropathy, leg cramps, blurred vision, arrhythmias, chest pain, hypotension, sensory neuropathy, psychiatric effects, hypersensivity reaction, proteinuria can occur. Please refer to the approved package insert for complete prescribing and toxicity information.

## 6.3 Drug Supply

Interferon Alpha-2a and Combivir are commercially available.

## 6.4 Chemotherapy Administration

6.4.1 EPOCH will be administered as follows:

Etoposide 50 mg/m$^2$/day given as a continuous 96 hr IV infusion on days 1-5.

Vincristine 0.4 mg/m$^2$/day given as a continuous 96 hrs IV infusion on days 1-5.

Doxorubicin 10 mg/m$^2$/day given as a continuous 96 hrs IV infusion on days 1-5.

Cyclophosphamide 750 mg/m$^2$ given IV on day 5. The infusion time for cyclophosphamide is 30 minutes.

Prednisone 60 mg/m$^2$ given orally on days 1-5.

6.4.2 Chemotherapy will be administered on a 21-28 day cycle for a minimum of 2 cycles beyond best response and a maximum of 6 cycles. "Best response" is the response achieved when 1 or more additional cycles of chemotherapy are given and no additional tumor shrinkage is noted. BSA will be recalculated at the beginning of each cycle. For patients with a stable disease, or progressive disease, no additional chemotherapy will be given. For patients with a PR or CR, 2 additional cycles will be given, but no more than 6 cycles of chemotherapy.

6.4.3 Administration of G-CSF: All patients will receive G-CSF at a dose of 5ug/kg subcutaneously daily beginning 24 hours after the administration of prednisone for 10 days beginning on day 6 or until the absolute neutrophil count has recovered to>4,000 cell/mm$^3$

## 6.5 Antiretroviral (ARV) Administration

Combivir 1 tablet po bid.

Interferon Alpha-2a 9 mU SQ qd.

## 6.6 Concurrent Medication

6.6.1 Antiretroviral Therapy: All patients will be given combination ARV medications after completing chemotherapy. For patients receiving antiretroviral therapy, prior to study initiation, for HIV, HBV, or HCV infection or other indication, these agents (other than AZT) may be continued at the investigator's discretion, but the antiviral drugs listed in 6.5 must be added to or substituted for the patient's current regimen at the completion of chemotherapy.

6.6.2 *Pneumocystis Carinii* Prophylaxis. All patients will receive prophylaxis with either co-trimoxozole, dapsone, or inhaled pentamidine.

6.6.3 Prevention of Tumor Lysis Syndrome (Cycle 1 only): It is recommended that patients with evidence of high number of circulating tumor cells (>50,000/ml) or

high tumor burden (bone marrow involvement, LDH>500) receive allopurinol, 600 mg 24 hours prior to the initiation of chemotherapy followed by 300 mg daily to continue for at least 7 days following administration of the first cycle of chemotherapy. Additional measures such as aggressive IV hydration and urinary alkalinization will be used at the discretion of the investigator.

6.6.4 All appropriate anti-infectives may be administered as clinically indicated.

## 6.7 CNS Prophylaxis

CNS prophylaxis will be left to the discretion of the investigator, but it is recommended that either of the following two regimens be provided:

a. Cytosine arabinoside, 50 mg administered intrathecally on days 1, 8, 15, and 22 of the first chemotherapy cycle. Please refer to the approved package insert for complete prescribing and toxicity information.

b. Methotrexate, 12 mg administered intrathecally on days 1, 8, 15, and 22 of chemotherapy cycle 1. Please refer to the approved package insert for complete prescribing and toxicity information.

## 6.8 Meningeal Lymphoma

Patients with positive CSF cytology will receive treatment as follows: AraC, 50 mg in preservative-free saline will be instilled using an intraventicular reservoir three times weekly until CSF cytology normalizes, then once weekly for 4 weeks, then on alternate weeks for 4 doses, then monthly for a total duration of intrathecal therapy of one year.

## 6.9 Radiotherapy

Whole brain radiotherapy is recommended for patients who present with neurologic signs and symptoms. It should be administered as soon as possible after diagnosis according to a standard protocol at each institution.

## 7.0 CLINICAL AND LABORATORY EVALUATIONS

### 7.1 Baseline/Pretreatment Evaluation

(See Appendix IV)

The following will be obtained no more than 30 days prior to the initiation of therapy:

7.1.1 Complete medical history to include:

7.1.1.1 Duration of HTLV-1 infection if known, birthplace, travel history, history of other complications of HTLV-1 infection.

7.1.1.2 Date of initial diagnosis of ATLL. A copy of the pathology report must be available in the medical record. Central pathological review is required within 30 days of study initiation. (Appendix III).

7.1.1.3 Presence or absence of "B"-symptoms (unexplained fevers, night sweats, involuntary weight loss greater than 10% normal body weight).

7.1.1.4 History of other symptoms related to ATLL or HAM.

7.11.5 History of drug allergies.

7.1.1.6 Medication list to include all antivirals, antibiotics, and prophylactic medications.

7.1.1.7 For $HIV^+$, date of diagnosis of HIV.

7.1.1.8 ACSB Donation after patient has provided consent.

7.1.2 Complete physical examination: includes Karnofsky performance score (see Appendix I), vital signs, weight, height, body surface area, neurologic examination, careful measurement of all palpable peripheral lymph nodes, and measurement of other sites of disease present on physical examination.

7.1.3 EKG

7.1.4 Urinalysis

7.1.5 Laboratory tests:

7.1.5.1 HTLV-1 serology by ELISA, confirmed by RIPA, PCR, or Western blot (GeneLabs Diagnostics, HTLV Blot 2.4).

7.1.5.2 Hematology: CBC, platelet count, and differential.

7.1.5.3 Blood chemistries: to include sodium, potassium, chloride, CO2, creatinine, calcium, phosphorus, uric acid, total bilirubin, AST, ALT, alkaline phosphatase, total protein, albumin, LDH.

7.1.5.4 T cell subsets (CD3, CD4, CD8)

7.1.5.5 Specimens sent for HTLV-1 proviral load, clonality, RNA, protein expression, HTLV-1 plasma viral load, apoptosis markers, and 3TC/ZDV sensitivity (See Appendix V).

7.1.5.6 Serum pregnancy test for women of childbearing age.

7.1.6 Staging Evaluation: The following studies will be done for baseline evaluation of extent of disease.

The Ann Arbor staging classification will be used (See Appendix II).

7.1.6.1 Chest x-ray.

7.1.6.2 CT or MRI scan of the chest, abdomen, and pelvis.

7.1.6.3 Bone marrow aspirate and biopsy (single core, at least 2.0 cm).

7.1.6.4 Lumbar puncture with routine studies and cytology.

7.1.6.6 Cytogenetics of malignant cells in blood, bone marrow, or tumor tissue.

## 7.2 Evaluations During Chemotherapy Treatment

(See Appendix IV)

7.2.1 Medical History.

7.2.2 Physical examination including Karnofsky performance score will be repeated on day 1 of each chemotherapy cycle. Disease measurable on physical examination should be measured in two dimensions.

7.2.3 CBC and differential will be repeated at the start of each chemotherapy cycle.

7.2.4 Electrolytes, creatinine, BUN, liver function tests (including total bilirubin, alkaline phosphatase, AST, ALT) and LDH will be obtained at the start of each chemotherapy cycle.

7.2.5 HTLV-1 proviral load, clonality, RNA, and proteins will be repeated on day 1 of each odd numbered chemotherapy cycle.

7.2.6 Specimens for HTLV-1 plasma virus load, and apoptosis markers, will be repeated on day 1 of each odd numbered chemotherapy cycle.

7.2.7 T cell counts will be repeated at the beginning of each odd numbered cycle of chemotherapy and at the conclusion of chemotherapy.

7.2.8 Restaging evaluation (CT or MRI, other studies as indicated) of all initial sites of measurable disease will be performed following completion of cycle 2 and cycle 4 (if cycles 3 and 4 given) and cycle 6 (if cycles 5 and 6 given) of chemotherapy.

7.2.9 Bone marrow biopsy should be performed after cycle 2 and cycle 4 (if cycles 3 and 4 given) and cycle 6 of chemotherapy (if cycles 5 and 6 given) if positive at the beginning of the study.

## 7.3 Evaluations During Antiretroviral (ARV) Treatment

(See Appendix IV)

7.3.1 Complete history each month.

7.3.2 Physical examination will be repeated monthly for psychiatric and thyroid disease.

7.3.3 CBC and differential will be repeated monthly.

7.3.4 Electrolytes, creatinine, BUN, liver function tests (including total bilirubin, alkaline phosphatase, AST, ALT) and LDH will be obtained monthly

7.3.5 HTLV-1 proviral load, clonality, RNA, protein expression and 3TC/ZDV sensitivity will be repeated every 3 months during ARV therapy.

7.3.6 HTLV-1 viral load will be repeated every 3 months during ARV therapy.

7.3.7 T cell counts will be repeated every 3$^{rd}$ month of ARV therapy.

7.3.8 Apoptosis markers will be repeated every 3$^{rd}$ month of ARV therapy

7.3.9 Restaging evaluation (CT or MRI, other studies as indicated) of all initial sites of measurable disease will be performed every 3 months of antiviral therapy.

7.3.10 Bone marrow biopsy will be repeated if positive on most recent evaluation.

## 7.4 Post-Treatment Evaluation

(See Appendix IV)

Patients will be seen every month for one year after completion of antiviral therapy, then every two months for one year, and then every 6 months for three additional years, to

document survival, performance status, and duration of response. CT or MRI scans will be repeated every 3 months during the first year of follow-up.

## 7.5 Early Discontinuation of Therapy

(See Appendix IV)

Patients going off study prior to completion of therapy will have a complete physical examination, and blood drawn for the following studies: CBC with differential and platelets, serum chemistries, calcium, T cell counts, HTLV-1 proviral load, clonality, RNA, and protein, HTLV-1 viral load, 3TC/ZDV sensitivity, and apoptosis markers.

# 8.0 DOSE MODIFICATION/TOXICITY MANAGEMENT

## 8.1 Chemotherapy Modifications: Hematologic Toxicity

8.1.1 If ANC<1000/mm$^3$ on day 1 of any treatment cycle, the next cycle will be delayed one week.

8.1.2 If after one week of delay, ANC>1000 with platelets >75,000, full dose therapy will be administered.

8.1.3 If ANC has not returned >1000 with platelets>75,000 after one week delay, drug doses will be modified on day 29 of the treatment cycle as follows:

| PLATELETS | ABSOLUTE NEUTROPHIL COUNT | | |
|---|---|---|---|
| | >1000/mm$^3$ | 500-1000/mm$^3$ | <500/mm$^3$ |
| >75 K | Full-dose | 50% cyclophosphamide<br>50% doxorubicin<br>50% etoposide | Hold treatment |
| 50-75 K | 50% cyclophosphamide<br>50% doxorubicin<br>50% etoposide | 50% cyclophosphamide<br>50% doxorubicin<br>50% etoposide | Hold treatment |
| <50 K | Hold treatment | Hold treatment | Hold treatment |

8.1.4 Patients unable to receive chemotherapy after a one-week delay will be followed with weekly CBC. If ANC>500 and platelets>50,000, these patients will be treated with 50% doses of cyclophosphamide, doxorubicin, and etoposide.

8.1.5 If ANC has not returned to >500 and platelets>50,000 by day 43, the patient will be withdrawn from the study.

8.1.6 If a nadir ANC count is obtained that is <500, then cyclophosphamide dose should be decreased by 25% on all subsequent chemotherapy cycles.

**8.2 Chemotherapy Modifications: Non-Hematologic Toxicity**

Any patients experiencing any grade 4 toxicity other than hematologic or infection will have no further therapy on this protocol.

8.2.1 Dose adjustment for hyperbilirubinemia

8.2.1.1 Bilirubin 1.2 to 3.0 mg/dl, reduce doxorubicin and vincristine doses by 50%.

8.2.1.2 Bilirubin 3.1-5.0 mg/dl, reduce doxorubicin and vincristine doses to 25% of full dose.

8.2.1.3 Bilirubin >5.0 mg/dl, doxorubicin and vincristine should not be administered.

8.2.1.4 If bilirubin>3.0 mg/dl for more than 30 days and is not associated with the presence of hepatic involvement with ATLL, the patient will be removed from the study.

8.2.2 Cardiac toxicity: If clinical findings suggesting congestive heart failure are present, doxorubicin will be discontinued and evaluation by MUGA will be performed.

8.2.3 Neutrotoxicity: vincristine will be discontinued permanently for peripheral neuropathy of grade 3 or more.

**8.3 ARV Toxicity Management**

8.3.1 For Grade 3 fever, fatigue, anorexia, mood disturbance.

8.3.1.1 For any of these toxicities, IFN should be held until the toxicity grade returns to <Grade 2 or baseline. IFN should be restarted at 5 mU/day.

8.3.1.2 If any of these toxicities persists for more than 14 days or recurs despite the dose reduction, then all study medications will be permanently discontinued.

8.3.2 For Grade 3 anemia, neutropenia, or thrombocytopenia

8.3.2.1 For any of these toxicities, IFN and combivir should be held until the toxicity grade returns to <Grade 2 or baseline. IFN should be restarted at 5 mU/day and combivir bid.

8.3.2.2 If any of these toxicities persists for more than 14 days or recurs despite the dose reduction, then all study medications will be permanently discontinued.

8.3.3 Hepatotoxicity

8.3.3.1 For Grade 3 elevations in AST (SGOT) or ALT (SGPT), all study medications will be held until the toxicity grade returns to <Grade 2 or baseline.

8.3.3.2 IFN should be restarted at 5 mU/day and combivir bid.

8.3.3.3 If ≥Grade 3 elevations in AST (SGOT) and ALT (SGPT) recur, all study medications should be held once again until the toxicity returns to <Grade 2 or baseline. IFN should be restarted at 1 mU/day and combivir bid.

8.3.3.4 If ≥Grade 3 elevations in AST (SGOT) and ALT (SGPT) recur a third time, then all study medications will be permanently discontinued.

8.3.4 For all other toxicities ≥Grade 3 and Grade 4 anemia, neutropenia, or hepatotoxicity, the following will be done:

8.3.4.1 All study medications will be held until the toxicity Grade returns to <Grade 2 or baseline. IFN should be restarted at 5 mU/day and combivir bid.

8.3.4.2 Blood transfusion and rEPO may be used for anemia ≥Grade 2 at the discretion of the investigator.

8.3.4.3 G-CSF may be used for neutropenia ≥Grade 3, at the discretion of the investigator.

8.3.4.4 If any of these toxicities persists for more than 30 days despite the interruption of all study medications or recurs despite a dose reduction of all study medications, then all study medications will be permanently discontinued.

8.3.5 For all other ≥Grade 4 toxicity, all study medications will be permanently discontinued.

## 9.0 CRITERIA FOR TREATMENT DISCONTINUATION

### 9.1 Patient Withdrawal

After enrollment the patient will be permanently withdrawn from study treatment for any of the following reasons:

9.1.1 Patients developing a life threatening infection who are in the chemotherapy portion of the protocol will have chemotherapy interrupted until the infectious process has cleared. The subject will be withdrawn from the study treatment only if chemotherapy has been held for more than six weeks.

9.1.2 Chemotherapy delays for more than six weeks, for any reason.

9.1.3 Severe toxicities as previously outlined.

9.1.4 Progressive ATLL after at least 2 cycles of chemotherapy and 1 cycle of antiviral therapy.

9.1.5 Voluntary withdrawal.

9.1.6 The investigator has the right to remove subjects from study for clinical reasons which he or she believes to be life threatening or resulting in significant morbidity to the subject.

## 10.0 REPORTING OF ADVERSE EVENTS

This study will utilize the CTCAE version 3.0 for Common Terminology Criteria for Adverse Event reporting. A copy of the CTCAE version 3.0 should be available at your institution. It can be downloaded from the CTEP home page. All appropriate treatment areas should have access to a copy of the CTCAE version 3.0. The documents "NCI Guidelines: Adverse Event Reporting Requirements for NCI Investigational Agents" (sections 2 and 3) clearly outline reporting criteria.

This study will be monitored by the Clinical Data Update System (CDUS). Cumulative CDUS data will be submitted quarterly to CTEP by electronic means. Reports are due January 31, April 30, July 31, and October 31.

### 10.1 Classification of Adverse Events by Severity and Relationship to Study Drug

**ADVERSE EVENT** – Any unfavorable and unintended sign (including an abnormal laboratory finding), symptom or disease temporally associated with the use of a medical

treatment of procedure regardless of whether it is considered related to the medical treatment or procedure (attribution of unrelated, unlikely, possible, probable, or definite).

**LIFE-THREATENING ADVERSE EVENT** – Any adverse event that places the patient or subject, in view of the investigator, at immediate risk of death from the reaction.

**SERIOUS ADVERSE EVENT (SAE)** – Any adverse event occurring at any dose that results in any of the following outcomes: death, a life-threatening adverse event, inpatient hospitalization or prolongation of existing hospitalization, a persistent or significant disability/incapacity, or a congenital anomaly/birth defect. Please note for **hospitalization** – All hospitalizations (or prolongation of existing hospitalization) for medical events equivalent to CTCAE Grade 3, 4, 5 must be reported regardless of the requirements for phase of study, expected or unexpected, and attribution. For example, do not report an admission for pharmacokinetic sampling, but do report an admission for a myocardial infarction.

**TOXICITY** – Toxicity is a term NOT clearly defined by regulatory organizations. Toxicity has been described as an adverse event that has an attribution of possibly, probably or definitely related to investigational treatment. To minimize confusion the NCI would recommend that the term toxicity NOT be utilized for adverse event reporting purposes.

**UNEXPECTED ADVERSE EVENT** – Any adverse event that is not listed in the NCI Agent Specific Expected Adverse Event List. This list is updated electronically in real time.

**ADVERSE EVENT EXPEDITED REPORTING SYSTEM (AdEERS)** (formerly known as Adverse Drug Reaction Reporting) – An electronic system for expedited submission of adverse event reports.

**ATTRIBUTION** – The determination of whether an adverse event is related to a medical treatment or procedure. Attribution categories:

Definite – The adverse event is *clearly related* to the investigational agent(s).
Probable – The adverse event is *likely related* to the investigational agent(s).
Possible – The adverse event *may be related* to the investigational agent(s).
Unlikely – The adverse event *is doubtfully related* to the investigational agent(s).
Unrelated – The adverse event *is clearly NOT related* to the investigational agent(s).

### 10.2 Adverse Events With Commercial Agents

The following adverse reactions must be reported to the AMC, the NCI and the local IRB in the manner described below.

Commercial Agents: Combivir, Interferon Alpha-2a, Doxorubicin, Vincristine, Etoposide, Prednisone, Cyclophosphamide, ARA-C, Methotrexate and Filgrastim.

Any unexpected (not listed in the package label), life threatening (Grade 4) or unexpected, fatal (Grade 5) adverse event with an attribution of possible, probable or definite should be reported in ten (10) working days. The AE should be reported on the FDA Form 3500 MedWatch (available from the FDA – www.fda.gov/medwatch).

The completed form should be forwarded to the FDA:

MedWatch
5600 Fishers Lane
Rockville, Maryland 20852-9787
Or fax to 1-800-332-0178

A copy should be forwarded to the NCI:
Investigational Drug Branch
P.O. Box 30012
Bethesda, Maryland 20824
Or fax to 301-402-1584

## 11.0 EVALUATION OF RESPONSE

All patients will be evaluated for clinical response by physical examination following each chemotherapy cycle and by imaging studies at the conclusion of the second cycle of chemotherapy and after the fourth cycle, if given, and after the sixth cycle if given. Complete re-staging will also be performed every 3 months during antiviral therapy and every 3 months during the first year of follow-up after completion of therapy.

### 11.1 Response Assessment

Response is assessed on the basis of clinical, radiologic, and pathologic (i.e. bone marrrow) criteria.

1. CT scans remain the standard for evaluation of nodal disease. Thoracic, abdominal, and pelvic CT scans will be performed for staging even if those areas were not initially involved because of the unpredictable pattern of recurrence in NHL.
2. A bone marrow aspirate and biopsy should only be performed to confirm a CR if they were initially positive or if it is clinically indicated by new abnormalities in the peripheral blood counts or blood smear.

## 11.2 Definition of Response

11.2.1 Complete Response (CR)

1. Complete disappearance of all detectable clinical and radiographic evidence of disease and disappearance of all disease-related symptoms if present before therapy, and normalization of those biochemical abnormalities (e.g. lactate dehydrogenase (LDH) or calcium) definitely assignable to ATLL.
2. All lymph nodes and tumor masses must have disappeared or regressed to normal size (less than or equal to 1.5 cm in their greatest transverse diameters for nodes >1.5 cm before therapy).

   Previously involved nodes that were 1.1 to 1.5 cm in their greatest transverse diameter before treatment must have decreased to less than or equal to 1 cm in their greatest transverse diameter after treatment, or by more than 75% in the sum of the products of the greatest diameters (SPD).
3. The spleen, if considered enlarged before therapy on the basis of a CT scan, must have decreased in size and must not be palpable on physical examination.

   Similarly, other organs considered to be enlarged before therapy due to involvement by ATLL, such as liver and kidneys, must have decreased in size.
4. If the bone marrow was involved by ATLL before treatment, the infiltrate must be cleared on repeat bone marrow aspirate and biopsy of the same site.
5. No new sites of disease.

11.2.2 Clinical Complete Response (CCR): Clinical complete responders will be grouped with complete responders if they qualify for inclusion by meeting all of the following criteria:

1. No abnormal palpable lymph nodes. In the event there is a suspicious or equivocal palpable lymph node, a negative biopsy is required.
2. No "B" symptoms (unexplained fevers, night sweats, involuntary weight loss greater than 10% normal body weight)
3. Chest radiograph and/or CT chest scan must be normal or, if mediastinal widening persists, the abnormal extra width must be diminished by at least 50% compared with pretreatment radiographs and there must have been no progression for eight weeks or more after the completion of therapy.
4. Gallium scan, if initially positive, must become negative or, if abnormal, there must have been no progression for eight weeks or more after the completion of therapy.
5. Residual opacified lymph nodes must return to normal as demonstrated by appearance on plain film of the abdomen or repeat lymphangiogram. If abnormal, there must have been at least a 50% reduction in the abnormal enlargement of the lymph nodes and there must have been no change in size for at least eight weeks after the completion of therapy. Architecture should be ignored for this type of evaluation.

6. CT abdomen/pelvic scan should be normal, or if abnormal, at least an 80% reduction from the initial abnormal enlargement of the measurable lesions should be seen and maintained for at least eight weeks after the completion of therapy.

11.2.3 Complete Response/Unconfirmed (CRu) Response includes those patients who fulfill criteria 1 through 4 above, but with one or more of the following features:

1. A residual lymph node mass greater than 1.5 cm in greatest transverse diameter that has regressed by more than 75% in the SPD. Individual nodes that were previously confluent must have regressed by more than 75% in their SPD compared with the size of the original mass.

2. Indeterminant bone marrow (presence of lymphoid aggregates without cytologic or architectural atypia)

11.2.4 Partial Response requires the following:

1. At least 50% decrease in SPD of the six largest dominant nodes or masses. These nodes or masses should be selected according to the following features: (a) they should be clearly measurable in at least two perpendicular dimensions, (b) they should be from disparate regions of the body as possible, and (c) they should include mediastinal and retroperitoneal areas of disease whenever these sites are involved.
2. No increase in the size of the other nodes, liver, or spleen.
3. Splenic and hepatic nodules must regress by at least 50% in the SPD.
4. With the exception of splenic and hepatic nodules, involvement of other organs is considered assessable and not measurable disease.
5. Bone marrow assessment is irrelevant for determination of a PR because it is assessable and not measurable disease; however if positive, the degree of marrow involvement should be specified.
6. No new sites of disease.

11.2.5 **Stable disease** is defined as less than a PR (see above) but is not progressive disease (see below)

11.2.6 **Progressive disease** (PD, non-responders) requires the following:

1. 50% increase from nadir in the SPD of any previously identified abnormal node for PD or non-responders.
2. Appearance of any new lesion during or at the end of therapy.

11.2.7 Recurrent disease is defined as the appearance of tumor following documentation of a complete remission.

11.2.8 Time to response is defined as time from the first dose of chemotherapy until documentation of first response.

11.2.9 Time to progression is defined as time from initiation of chemotherapy to documentation of first progression.

11.2.10 Response duration is defined as the time from first documentation of response to documentation of first progression.

## 12.0 STATISTICAL CONSIDERATIONS

### 12.1 Sample Size Estimation and Accrual

A two-stage Simon design will be adopted to allow early termination for treatment inefficacy. It is assumed that the level of response below which one considers the regimen insufficiently active for further pursuit is 10%, and that the level at which one would have considerable interest in future testing is 30%. To test the null hypothesis that the response rate is less than equal to 10% versus the alternative hypothesis that it is at least 30% with a significance level of 5% and power of 80% will require 29 patients. Ten patients will be enrolled during the first stage. An interim analysis will be conducted after all 10 patients have completed chemotherapy. If at least 2 objective responses are observed, the study will proceed to the second stage and an additional 19 patients will be enrolled into the study. Sufficient evidence of the treatment will be concluded if at least 6 out of 29 patients exhibit response. Assuming a 10% dropout rate, 32 subjects will be required. It is estimated that this study will accrue patients at a rate 16 patients per year.

### 12.2 Statistical Analysis Plan

Binomial proportions and their 95% confidence intervals will be used to estimate the response rates to therapy. Logistic regression analyses will be used to evaluate baseline characteristics and other covariates with response. The Kaplan-Meier method will be used to evaluate the response duration. The Cox proportional hazards model will be used to evaluate baseline characteristics and other covariates with respect to response duration. Analyses of variance methods will be used to evaluate the effects of treatment and time on the viral load measurements, as well as measurement of viral transcripts, Tax protein, p53, phosphorylated p53, bcl-xL, and caspase 3 cleavage products. The incidence of toxicities will be estimated using the binomial proportion and its 95% confidence interval. A proportional hazards analysis with viral load measures as time dependent covariates will be used to evaluate the effects of these measures on duration of response.

### 12.3 Data Safety Monitoring

This protocol will follow the AMC's policy for data monitoring (See Appendix VIII).

## 13.0 ETHICAL AND REGULATORY CONSIDERATIONS

### 13.1 Informed Consent

The principles of informed consent described in Food and Drug Administration (FDA) regulations (21 CFR part 50) must be followed. IRB approval of the protocol and the informed consent form must be given in writing.

The sponsor must receive a copy of the letter of approval from the IRB, which specifically approves the protocol and informed consent, before patient enrollment. The IRB must also approve any significant changes to the protocol and documentation of this approval must be sent to the sponsor. Records of all study review and approval documents must be kept on file by the investigator and are subject to FDA inspection during or after completion of the study. Adverse events must be reported to the IRB. The IRB should receive notification of completion of the study and final report within three months of study completion and termination. The investigator will maintain and accrue and complete record of all submissions made to the IRB, including a list of all reports and documents submitted.

### 13.2 Women and Minorities

This is a study being conducted by the NCI sponsored AIDS Malignancy Consortium (AMC). As part of their contractual obligations, each participating site within the AMC and the AMC as a whole are required to assure that the participation of women and minority subjects reflects the percentage representation of these populations in their geographic region and, for the AMC, the United States as a whole. As such, it is expected that the representation of subjects on this trial will reflect the constitution of the respective populations.

## 14.0 REFERENCES


1. Takatsuki, K., Adult T-cell leukemia. Int Med. 34:947-952, 1995
2. Gessain, A., Mahieux, R., de The, G., Genetic variability and molecular epidemiology of human and simian T cell leukemia/lymphoma virus type I. J AIDS 13: Suppl 1:S132-145, 1996.
3. Manns A., Hisada, M., La Grenade, L. Human T-lymphotropic virus type I infection. Lancet 353:1951-1958, 1999.
4. Franchini, G. Molecular mechanisms of human T cell leukemia/lymphotropic virus type 1 infection. Blood 86:3619-3639, 1995.
5. Hollsberg, P. Mechanisms of T-cell activation by human T-cell lymphotropic virus type I. Microb Molec Biol Rev 63:308-333, 1999.
6. Mesnard, J-M., Devaux, C. Multiple control levels of cell proliferation by human T-cell leukemia virus type 1 tax protein. Virol 257:277-284, 1999.
7. Shimoyama, M. Diagnostic criteria and classification of clinical subtypes of adult T-cell leukaemia-lymphoma: a report from the Lymphoma Study Group (1984-87). Br J. Haematol 79:428-437, 1991.
8. Nagai, M., Usukiu, K., Wataru, M., et al., Analyis of HTLV-I proviral load in 202 HAM/TSP pateints and 243 asymptomatic HTLV-I carriers: high proviral load strongly predisposes to HAM/TSP. J. NeuroVirology. 4:685-593, 1998.
9. Ohshima, K., Suzumiya, J., Izumo, S., et al., Detection of human T-lymphotropic virus type-I DNA and mRNA in the lymph nodes; using polymerase chain reaction in situ hybridization (PCR/ISH) and reverse transcription (RT-PCR/ISH). Int J. Cancer 66:18-23, 1996.

10. Yoshida, M., Seiki M., Yamaguchi, K., Takatsuki, K. Monoclonal integrationof human T-cell leukemia provirus in all primary tumors of adult T-cell leukemia suggests causative role of human T-cell leukemia virus in the disease. Proc. Natl. Acad. Sci. U.S.A. 81:2534-2538 , 1984.
11. Wattel, E., Cavrois, M., Gessain, A., Wain-Hobson, S. Clonal expansion of infected cells: a way of life for HTLV-I. J AIDS 13:Supple1:S92-99, 1996.
12. Bazarbachi, E., El-Sabban, M. E., Nasr, R., et al., Arsenic trioxide and Interferon-Alpha-2a synergize to induce cell cycle arrest and apoptosis in human T-cell lymphotropic virus type I-transformed cells. Blood 93:278-283, 1999.
13. Lymphoma Study Group. Major prognostic factors of patients with adult T-cell leukemia-lymphoma: a cooperative study. Leuk Res 15:81-90, 1991.
14. Bunn, P. A., Schechter, G. P., Jaffe, E,., et al. Clinical course of retrovirus-associated adult T-cell lymphoma in the United States. N Engl. J. Med. 309:257-264, 1983
15. Lofters, W., Campbell, M., Gibbs, W. N., Cheson, B. D. 2'Deoxycoformycin therapy in adult T-cell leukemia/lymphoma. Cancer 60:2605-2608, 1987.
16. Taguchi, H., Kinoshita, K. I., Takatsuki, K., et al. An intensive chemotherapy of adult T-cell leukemia/lymphoma: CHOP followed by etoposide, vindesine, ranimustine, and mitoxantrone with granulocyte colony-stimulating factor support. J. AIDS 12:182-186, 1996.
17. Gill, P. S., Harrington, W., Kaplan, M. H., et al. Treatment of adult T-cell leukemia-lymphoma with a combination of Interferon Alpha-2a and zidovudine. N Engl. J Med 332: 1744-1748, 1995.

18. Hermine, O., Bouscary, D., Gessain, A., et al., Treatment of adult T-cell leukemia-lymphoma with zidovuine and Interferon Alpha-2 a. N Engl J. Med 332:

19. Dega, H., Chosidow, O, Charlotte, F., et al., Unsuccessful association of zidovudine and Interferon Alpha-2a for acute adult T-cell leukemia lymphoma. Dermatology 198:103-105, 1999.

20. Waldmann, T. A., Goldman, C. K., Bongiovanni, K. F., et al., Therapy of patients with human T-cell lymphotropic virus I-induced adult T-cell leukemia with anti-Tac, a monoclonal antibody to the receptor for interleukin-2. Blood 72:1805-1816, 1988.

21. Taylor, G. P., Hall, S. E., Navarrete, S., et al., Effect of lamivudine on human T-cell leukemia virus type 1 (HTLV-1) DNA copy number, T-cell phenotype, and anti-Tax cytotoxic T-cell frequency in patients with HTLV-1-associated myelopathy J. Virol. 1999 73: 10289-10295.

22. Journal of Clinical Oncology, Vol 18, Issue 21 (November), 2000:
3633-3642 Role of a Doxorubicin-Containing Regimen in Relapsed and Resistant Lymphomas: An 8-Year Follow-Up Study of EPOCH
By Martin Gutierrez, Bruce A. Chabner, Debra Pearson, Seth M. Steinberg, Elaine S. Jaffe, Bruce D. Cheson, Antonio Fojo, Wyndham H. Wilson

23. Wilson WH, Bryant G, Bates S, et al: EPOCH chemotherapy: Toxicity and efficacy in relapsed and refractory non-Hodgkin's lymphoma. J Clin Oncol 11: 1573-1582, 1993

24. Little RF, Pearson D, Steinberg S, et al.: Dose-adjusted EPOCH chemotherapy (CT) in previously untreated HIV-associated non-Hodgkin's lymphoma (HIV-NHL). American Society of Clinical Oncology, 35th Annual Meeting. Atlanta; 1999: Abstract 33

## Appendix I: Karnofsky Performance Scale

| | | |
|---|---|---|
| Able to carry on normal activity; no special care is needed | **100** | Normal; no complaints; no evidence of disease |
| | **90** | Able to carry on normal activity; minor signs or symptoms of disease |
| | **80** | Normal activity with effort; some signs of symptoms of disease |
| Unable to work, able to live at home and care for most personal needs; a varying amount of assistance is needed | **70** | Cares for self; unable to carry on normal activity or to do active work |
| | **60** | Requires occasional assistance but is able to care for most of his needs |
| | **50** | Requires considerable assistance and frequent medical care |
| Unable to care for self; requires equivalent of institutional or hospital care; disease may be progressing rapidly | **40** | Disabled; requires special care and assistance |
| | **30** | Severely disabled; hospitalization is indicated although death is not imminent |
| | **20** | Very sick; hospitalization necessary; active supportive treatment is necessary |
| | **10** | Moribund; fatal processes progressing rapidly |
| | **0** | Dead |

## Appendix II: Ann Arbor Staging Criteria

| STAGE | DESCRIPTION |
|---|---|
| STAGE I | Involvement of a single lymph node region (I) or of a single extralymphatic organ or site (IE) |
| STAGE II | Involvement of two or more lymph node regions on the same side of the diagphragm (II), or localized involvement of extralymphatic organ or site and of one or more lymph node regions on the same side of the diagphragm (IIE) |
| STAGE III | Involvement of lymph node regions on both sides of the diaphragm (III), which may also be accompanied by localized involvement of extralymphatic organ or site (IIIE) or by involvement of the spleen (IIIS), or both (IIISE) |
| STAGE IV | Diffuse or disseminated involvement of one or more extralymphatic organs or tissues with or without associated lymph node enlargement |

A Absence of systemic symptoms

B Presence of one or more general symptoms: (1) unexplained weight loss of more than 10% of the body weight in the 6 months before admission; (2) unexplained fever with temperature above 38C; (3) night sweats

---

Notes:

1. The lymphatic structures are defined as the lymph nodes (N), spleen (S), thymus, Waledeyer's ring, appendix, and Peyer's patches

2. The reasons for classifying the patient as stage IV is defined further by defining sites by symbols:

| | |
|---|---|
| H-Liver | L-Lung |
| M-Marrow | O-Bone |
| P-Pleura | D-Skin |

3. Liver involvement is always considered Stage IV disease, as is bone marrow involvement away from a site of an involved lymph node.

**Appendix III: Central Pathology Review**

Diagnostic slides will be centrally reviewed by an expert panel of hematopathologists. Within 30 days of registration, diagnostic slides, a copy of the original pathology report, 10 unstained slides or the diagnostic paraffin block and forwarded to:

Immunopathology
Starr 715
New York Presbyterian Hospital
Cornell Campus
525 East 68th Street
New York, NY 10021

Contact: Dr. Amy Chadburn
Phone: 212-746-2442
Fax: 212-746-8173
Monday thru Friday, 8:00 a.m. – 6:00 p.m. Eastern Time

Specify in the paperwork that the sample is from AMC for Central Pathology

**Records of Specimens:** Specimens should be accompanied by the specimen procurement form (RF36) and Specimen Shipping Log (RF31). Copies of these forms should also be forwarded to the AMC Operations Center at UAB.

## Appendix IV: Schedule Of Evaluations

| | Baseline/ Pretreatment | Chemo Therapy Day 1 | Antivirals Day 1 each cycle | Antivirals Day 1 every third cycle | Post Therapy Each Visit | Post Therapy Every 3 Months, Yr 1 |
|---|---|---|---|---|---|---|
| Complete History | X | X | X[1] | | X | |
| Physical Exam | X | X | X[1] | | X | |
| CBC, Diff, Platelets | X | X | X[1] | | X | |
| Serum Chemistries | X | X | X[1] | | X | |
| T-Cell Subsets | X | Odd Number Cycles | | X[1] | | X |
| Collect 30 ml Blood [2] | X | Odd Number Cycles | | X[1] | | X |
| EKG | X | | | | | |
| CXR | X | | | | | |
| Serum Pregnancy | X | | | | | |
| CT or MRI | X | At completion of 2nd, 4th and 6th cycle | | X[1] | | X |
| Bone Marrow | X | If positive at baseline, at completion of 2nd and 4th cycles | | | | |
| Lumbar Puncture | X | | | | | |
| ACSB Donation[3] | X | | | | | |

[1] Antiretroviral therapy is a 28 day cycle.
[2] The 30 ml blood collection is for "HTLV-1 proviral load, RNA and protein expression, plasma virus load, apoptosis markers, and 3TC/ZDV sensitivity.
[3] ACSB Donation to be collected after patient has provided consent.

## Appendix V: Blood For HTLV-1 Proviral Load, Clonality, RNA & Protein Expression, Plasma Virus Load, & Apoptosis Markers

### COLLECTION

Collect three yellow top tubes (30 ml) of whole blood on the days when HTLV-1 proviral load, clonality, RNA and protein expression, plasma virus load, apoptosis markers, and 3TC/ZDV sensitivity are done (see Appendix IV: Schedule of Evaluations).

### SHIPPING

To ship bloods, place three yellow top tubes (30 ml) into a canister of a STP-100 SAF-T-PAK shipper (VWR# 11217-163) wrapping each tube in bubble wrap and using the absorbent paper at the bottom of the canister. Each sample tube should be labeled using a sharpie pen with the following information:

- Protocol #: AMC 033
- 9 digit Patient #
- Patient initials
- Date and time of collection
- Specimen type – "Whole Blood"
- Specimen purpose: Virologic assays

Place the lid on the canister and place it inside of the ambient SAF-T-PAK shipper indicating on packaging "Virologic Assays, Attention: Dr Ratner". Seal the ambient shipper with cellophane shipping tape. Label the ambient shipper with the "Infectious substance" diamond shaped label. On one side, in black marker write "INFECTIOUS SUBTANCE AFFECTING HUMANS (HTLV) UN2814, your name or name of responsible person, date of collection and 24 hour phone number of the person responsible for the package.

**Specimen Shipment.** Specimens may be shipped MONDAY through THURSDAY. All specimens should be shipped by overnight express at room temperature to:

Lee Ratner MD PhD
CSRB 10042 - Oncology
Washington University
4940 Parkview Pl
St Louis, MO 63110
TEL: (314) 362-8836
FAX: (314) 747-2797
Lratner@imgate.wustl.edu

Use FED-EX "Dangerous Goods" airway bills for shipping. FED-EX account for the shipment: 2075-8461-4. <u>It is only to be used for billing shipment of specimens to the lab where the sample</u>

is processed and/or stored. Call FED-EX at1-800-463-3339 and press 0. Ask for customer service "dangerous goods" department. A FED-EX representative will assist in the specific wording required on the airway bills for pick-up and delivery of "Dangerous Goods". Place the completed airway bill marked **"Priority Overnight"** and the typed "Shippers Declaration for Dangerous Goods" on top of the shipper box inside of a plastic FED-EX pouch. ***PLEASE DOUBLE CHECK PACKAGING OF SHIPPER AND DO NOT DEVIATE FROM REQUESTED LABELING.

**Please Note**: The shipper will be mailed back to the AMC site.

**The STP**-100 SAF-T-PAK shipper (VWR Cat #11217-163) is a complete kit w/all trappings, bubble wrap, adsorbent paper, labels, everything (but to reuse the shipper, you will need new labels, wrap, etc). There is a refurbishment kit w/extra b- wrap, adsorbent material (STP 102) (VWR Cat #11217-166) enough for 15 mailings.

**Record of Specimens.** Specimens should be accompanied by the Specimen Procurement Form (RF36) and Specimen Shipping Log (RF31). Copies of these forms should also be forwarded to the AMC Operations Center at UAB.

## Appendix VI: Informed Consent

### Consent To Be A Research Subject

### AMC 033 - PHASE II TRIAL OF INDUCTION THERAPY WITH EPOCH CHEMOTHERAPY AND MAINTENANCE THERAPY WITH COMBIVIR/INTERFERON ALPHA-2a FOR HTLV-1 ASSOCIATED T-CELL NON-HODGKIN'S LYMPHOMA

This consent form may contain words that you do not understand. Please ask the study doctor or the study staff to explain any words or information that you do not clearly understand.

**Background/Purpose**

You are invited to participate in a research study conducted by Lee Ratner, M.D. and/or colleagues. The overall purpose of this research is:

You are being asked to participate in this research study because you have been diagnosed with adult T cell leukemia/lymphoma that is related to your HTLV-1 (human T-cell leukemia virus type 1) infection. The purpose of this study is to determine if antiretroviral agents, drugs that block HTLV-1, if given in combination with chemotherapy, results in improved response rates. The study also aims to evaluate the effect of chemotherapy and antiretrovirals on immune function (the body's ability to fight infection) and viral load (the level of HTLV-1 in the bloodstream.)

Interferon (IFN) Alpha-2a will be used daily after chemotherapy is completed. It has been used extensively in many clinical disorders, including HTLV-1 infected subjects. The other antiretroviral agent, combivir is approved for HIV infection, but has not yet been used in HTLV-1 infections. The combination of chemotherapy with antiretrovirals has not yet been administered to patients with HTLV-1-related adult T cell leukemia/lymphomas like yours, and is therefore investigational.

A total of 32 patients are anticipated for enrollment in this multicenter study.

**Procedures**

If you agree to participate in this study, the following will occur:

1. Your medical history will be reviewed and you will have a complete physical examination. You will be required to have certain tests performed, which are considered standard care for your NHL. These tests include a chest x-ray, CT scan or other imaging study as well as various blood tests and a urinalysis. A biopsy of your tumor to determine its cell type, a lumbar puncture, also known as a spinal tap, and at least 2 more bone marrow biopsies will be required, if positive at the beginning of the study. These procedures will be fully explained to you and a separate consent will be obtained for each procedure.

If these tests determine that you are eligible to participate, you will have a central venous tube (catheter) placed in a vein for the intravenous administration of chemotherapy. Treatment will take place in the inpatient unit. During the infusions your vital signs (blood pressure, pulse, respiration and temperature) will be monitored frequently. The infusion should take approximately 5 days. Chemotherapy drugs etoposide, vincristine, and doxorubicin will be given continuously in a vein for the first 5 days of each chemotherapy cycle. Cyclophosphamide will be given over 30 min in a vein on the 5$^{th}$ day of each chemotherapy cycle. Prednisone tablets will be given orally each day for the first five days of each chemotherapy cycle. Each chemotherapy cycle will be 21-28 days, although chemotherapy is only given during the first 5 days of each cycle.

Upon completion of chemotherapy, you will receive combivir orally two times daily and IFN Alpha-2a subcutaneously daily. You will be instructed by the clinic staff how to administer IFN Alpha 2a to yourself. This is known as the maintenance phase of the study.

2. At least 2 cycles of chemotherapy will be given. Exactly how many cycles you will receive is dependent upon how rapidly you respond to therapy, but the maximum number of cycles is 6.

3. You will be treated with a medication called G-CSF (filgrastim) to help increase your white blood cell count while receiving chemotherapy. G-CSF will be given by injection just under the skin daily beginning 24 hours after the administration of prednisone for 10 days beginning on days 6 through 16. You will be instructed by hospital staff how to administer G-CSF to yourself. G-CSF will not be supplied by the study.

4. You will have 30cc (6-8 samples) of blood drawn on eight occasions to assess your immune function against HTLV-1 and the levels of HTLV-1 in your blood. These studies are primarily to guide future treatment of others and are unlikely to have an impact on your therapy.

5. Imaging studies will be done at every second chemotherapy cycle to evaluate your response to treatment unless your NHL is measurable by physical exam. Lumbar puncture and bone marrow biopsy will be repeated only if these were positive at the beginning of the study.

6. 1-5 tablespoonful(s) of blood will be collected every 3-4 weeks during treatment and every 4 weeks during the maintenance phase for laboratory studies on the first day each cycle. A physical exam will be done at every visit.

7. If your doctor determines that there is a high risk of spread of your lymphoma to the membranes surrounding the brain and spinal cord (meningitis) you will have preventive chemotherapy injected directly into the spinal fluid once weekly for the first four weeks of chemotherapy. This is standard therapy for this type of lymphoma and is done by performing a lumbar puncture.

8. If the initial lumbar puncture shows lymphoma cells in your spinal fluid, more extensive treatment will be required to eliminate the disease from this location. The treatment will require frequent injections of chemotherapy directly into the spinal fluid and radiation therapy to the brain. This is standard therapy for meningeal lymphoma and will be carefully explained to you by your doctor.

9. Follow up visits will occur every 4 weeks for one year after completion of maintenance therapy and then every 2 months for an additional year to evaluate survival. Follow up visits will include a physical exam and some blood drawing for immune function tests. The patient will be monitored every 6 months for 3 additional years after the two-month intervals.

10. If you discontinue study participation early, you will have a complete physical examination and blood collected for laboratory tests.

11. When chemotherapy is started, you may be treated with allopurinol, a drug for the prevention of "tumor lysis syndrome", a condition caused by rapid release of cell material into the bloodstream as a result of tumor destruction from chemotherapy drugs. Allopurinol helps to reduce the chances of this material reaching dangerously high levels in your blood.

**Risks and Discomforts**

There are certain risks and discomforts that may be associated with this research. They include:

Cyclophosphamide

Likely: Common side effects of cyclophosphamide include nausea, vomiting, lowered white blood cell counts (which can increase the risk of infections), lowered platelet (a type of blood cell) counts (which can cause increased bleeding).
Less Likely: Rarely, sterility (inability to conceive a child) in men and amenorrhea (temporary or permanent cessation of regular menstrual periods) in women may occur. Bladder irritation, including blood in the urine have been known to occur

Etoposide

Likely: The most common side effect of etoposide is lowered white blood cell counts (which can increase the risk of infections).
Less Likely: Constipation, diarrhea, painful swallowing, aftertaste, abdominal pain, mouth sores, and loss of appetite have been reported with the use of etoposide. Liver function abnormalities are seen primarily with higher doses. Low blood pressure and other allergic symptoms are associated with rapid infusion.

Doxorubicin

Likely: Doxorubicin is commonly associated with low blood counts (which can lead to infections or require blood transfusions), hair loss, inflammation of the mouth (also known as stomatitis), nausea and vomiting. Doxorubicin may also cause lowered white blood cells and platelets. If doxorubicin

accidentally leaks from the intravenous line, it can cause severe irritation at the site of administration. If the cumulative dose (total amount of drug given over the entire course of treatment) reaches or exceeds 500 mg/m$^2$, the risk of developing cardiomyopathy, a condition that causes a debilitating decrease in heart function, increases considerably.

Vincristine
Likely: Vincristine may cause peripheral neuropathy, a condition characterized by reduced or lost reflexes, numbness and tingling in the arms and legs, as well as muscle aches and weakness.
Less Likely: Constipation, abdominal cramps and obstruction have been associated with vincristine. Low blood counts are a known side effect but not to the degree caused by cyclophosphamide and doxorubicin. Like doxorubicin, if vincristine accidentally leaks from its intravenous line, it can cause severe irritation at the site of administration.

Prednisone

Likely: Prednisone causes minimal side effects with short-term use. Common side effects are mood swings and insomnia. Prolonged use of prednisone can cause high blood pressure, high blood sugar levels, myopathy (muscle abnormalities), osteoporosis (weakening of the bones), inflammation of the pancreas, and water retention, which can cause swelling.

G-CSF

Likely: The most common side effects of G-CSF are aching bones and high white blood cell counts.

Chemotherapy may cause suppression of the immune system, which could lead to infections.

IFN Alpha-2a

Likely: Dizziness, tiredness, fever, chills, rash, mouth soreness, nausea, vomiting, diarrhea, abdominal cramps, weight loss, metallic taste, lower white blood cell counts (that may cause infections), lower red blood cell counts (that may cause tiredness), lower platelet count (a blood cell that is needed for bleeding), sweating.

Rare: Headache, confusion, sleepiness, altered sensation, hair loss, dry skin, loss of appetite, mouth sores, liver function abnormalities, leg cramps, blurred vision, heart rhythm abnormalities, chest pain, low blood pressure, psychiatric effects, allergic reactions, or protein loss in the urine. Alpha Interferons, including IFN Alpha 2a, cause or aggravate fatal or life-threatening neuropsychiatric, autoimmune, ischemic, and infectious disorders. Patients will be monitored closely with periodic clinical and laboratory evaluations for these problems. Patients with persistently severe or worsening signs or symptoms of these conditions will be withdrawn from therapy. In many, but not all cases, these disorders resolve after stopping IFN Alpha-2a therapy.

Combivir is a combination of zidovudine and lamivudine.

Zidovudine

Likely: Headache, fever, rash, nausea, loss of appetite, diarrhea, pain, vomiting, lower red blood cell counts (that may cause tiredness), weakness, lower white blood cell counts (that may cause infections).

Less Likely: Tiredness, dizziness, difficulty sleeping, sleepiness, increased skin pigmentation, liver function abnormalities, weakness.

Lamivudine

Likely: Headache, difficulty sleeping, fatigue, pain, nausea, diarrhea, vomiting, loss of sensation, tingling, nasal symptoms, cough.

Rare: Dizziness, depression, fever, chills, rash, loss of appetite, abdominal pain, acid taste, elevated pancreatic or liver function tests, lower red blood cell counts (that may cause tiredness), weakness, lower white blood cell counts (that may cause infections), muscle aches, joint aches.

Blood drawing may cause some discomfort, bleeding or bruising where the needle enters the skin, and rarely, fainting or infection may occur.

**Combination Drug Therapy**
These drugs may cause more serious side effects than when given alone.

**New Findings**
You will be informed of any significant new findings developed during the course of participation in this research that may have a bearing on your willingness to continue in the study. The investigator may withdraw you from this research if circumstances arise which warrant doing so.

**Confidentiality**

Participation in research will involve a loss of privacy. Your research records will be handled as confidentially as possible. All research records will be coded. In order to verify the study data, representatives from the AMC, National Cancer Institute, the Food and Drug Administration, or a qualified representative of the drug manufacturer may need to review your records. No individual identities will be used in any reports or publications resulting from this study.

**Treatment and Compensation for Injury**
If you are injured as a result of being in this study, treatment will be available. The costs of such treatment may be covered by ______________________, depending on a number of factors. ______________________ does not normally provide any other form of compensation for injury. For further information you may call ___________________________________.

Pregnancy/Childbearing Potential

| **If you are a woman of childbearing potential, please read and acknowledge the following section.** |
|---|
| Some research medications or procedures can cause severe birth defects and mental retardation to a fetus (unborn baby). If you are pregnant, think you are pregnant, or if at any time during your study participation there is a lapse in your birth control procedures, it is important for you to notify the investigator immediately. It is important that you inform the study doctor if you are lactating female (actively breastfeeding).<br><br>If you participate in a study that includes a drug or procedure, you must be willing to: have a pregnancy test done at the discretion of the investigator before beginning your participation; avoid becoming pregnant during your participation; and agree to use a method of birth control that is acceptable with the investigator.<br><br>Your signature below indicates your agreement to comply fully with these requirements.<br><br>________________________________________<br>Signature Date |

Investigators to use at their discretion:

| **If you are a sexually active male, please read and acknowledge the following section.** |
|---|
| There is concern that some research medications or procedures may cause severe birth defects and/or mental retardation to a fetus (unborn baby).<br><br>If you participate in a study that includes a drug or procedure, you and your partner must be willing to use a method of birth control that is agreeable with the investigator.<br><br>Your signature below indicates your agreement to comply fully with these requirements.<br><br>________________________________________<br>Signature Date |

**Benefits**

The possible benefits to you and society from this research are:

Benefits of participating in this study include the possibility of a complete or partial response of your adult T-cell leukemia/lymphoma. However, it is not possible to predict whether or not any benefit will result from your taking part in this study, but knowledge may be gained that may benefit others.

YOUR PARTICIPATION IS VOLUNTARY and you may choose not to participate in this research study or withdraw your consent at any time. Your choice will not at any time affect the commitment of your health care providers to administer care and there will be no penalty or loss of benefits to which you are otherwise entitled. Other than non-participation in the research, available alternatives include: chemotherapy alone, antiviral therapy alone, a different combination of chemotherapy and antiviral therapy, antibodies or growth factors linked to toxins or radioisotopes, or arsenic.

**Alternatives**

Alternatives to participation in this study include standard chemotherapy with or without rituximab, investigational drugs, radiation or no treatment at all. Your physician will go over in detail all of your therapeutic options and answer all your questions regarding the treatment of your illness.

**Questions**

If you have any questions or concerns regarding this study, or if any problems arise, you may call the Principal Investigator at (Institution ). You may also ask questions or state concerns regarding your rights as a research subject to (____________________________________).

(Institution) investigators and their colleagues who provide services at (_______________) hospitals and facilities recognize the importance of your contribution to research studies that are trying to improve medical care. (Institution) investigators and their staffs will make every effort to minimize, control, and treat any complications that may arise as a result of this research. If you believe that you are injured solely as the result of the research question being asked in the study, please contact the Principal Investigator and/or the Chairman of the Human Studies Committee as stated above. (Institution) reserves the right to make decisions concerning payment for medical treatment for injuries solely and directly relating to your participation in biomedical or behavioral research.

**Costs**

Taking part in research studies may or may not lead to added costs to you or your insurance company. Please ask the investigator and/or your doctor about any added costs or insurance problems that might be associated or expected with participating in this particular study.

You will not be charged for any procedures or treatments that are being done only for research purposes.

You or your insurance company will, however, be charged for any other portion of your care that is considered standard care, this includes the costs of imaging studies, X-rays, routine blood tests, bone marrow biopsies, lumbar punctures, chemotherapy and its administration, G-CSF, antiretroviral medication and clinic visits. You will not be responsible for the cost of blood tests performed for measurement of viral load.

In the case of injury or illness resulting from this study, emergency medical treatment is available but will be provided at the usual charge. No funds have been set aside to compensate you in the event of injury. No payment is made to patients participating in this research study.

This research is not intended for the purpose of diagnosing or treating any medical problems not specifically stated in the purpose of the research. Participation in a research study does not take the place of routine physical examinations or visits to your personal physician.

**I have read this consent form and have been given the opportunity to ask questions. I will also be given a signed copy of this consent form for my records. I hereby consent to my participation in the research described above, titled: Chemo/Antiretroviral Therapy for HTLV-1 ATLL**

________________________________________

Parent or legal guardian's signature on participants' behalf if participant is less than 18 years of age or not legally competent. (Blood drawing only: Less than 17 years of age.)

________________________________________

Participant's Signature Date

____________________________________

Relationship to Child

Date______________

Informed Consent provided by:

________________________________________

Signature of Principal or Collaborating Investigator when informed consent responsibility is entrusted to a designee. (See HSC Guidelines on *Who May Obtain Consent to Participate in Research Activities*.)

________________________________________

Signature of Principal Investigator or Designee Date

## APPENDIX VII: GRID OF FORMS SUBMISSION TABLE

| | | Time Period (Study Therapy/Follow-up) | | | | |
|---|---|---|---|---|---|---|
| **Form #** | **Description** | **Baseline** | **Chemo Cycles (Day 1)** | **Antivirals (Monthly)** | **Post-therapy Follow-up** | **PRN** |
| **Registration** | | | | | | |
| RF01B | Registration Form | X | | | | |
| PR3301 | Eligibility Form | X | | | | |
| **Medical History** | | | | | | |
| PR3302 | HX HTLV-1 | X | | | | |
| RF02 | On Study Form | X | | | | |
| RF06 | Prior Antiretroviral Therapy | X | | | | |
| RF07 | Prior medications excluding antiretroviral Form | X | | | | |
| RF09 | Concomitant Medications | | X | X | X | |
| **Physical Examination** | | | | | | |
| RF20 | Vital Signs Form | X | X | X | X | |
| RF21 | Complete PE Form | X | X | X | X | |
| **Laboratory Tests** | | | | | | |
| RF22 | Hematology | X | X | X | X | |
| RF23 | Chemistries, LFTs | X | X | 3rd Month | X | |
| RF24 | T-Cell Lab Values | X | Every odd cycle | X<br>3rd Month | X<br>3rd Month | |
| RF25 | Urinalysis | X | | | | |
| RF27 | Abnormal Lab Values | X | X | X | X | |
| **Treatment** | | | | | | |
| PR3303 | Study Drug Therapy | | X | X | | |
| PR3304 | Chemotherapy Form | | X | | | |
| PR3305 | Antiretroviral Therapy Form | | | X | | |
| **Disease Evaluation** | | | | | | |
| RLY02 | Baseline Lymphoma Assessment Form | X | | | | |
| RLY03 | Lymphoma Disease and Activity Form | | End of cycle<br>2 / 4 / 6 | X<br>3rd Month | X<br>3rd Month | |
| **Toxicity** | | | | | | |
| RF28B | Reported Adverse Events | | X | X | | |
| **Specimens for Central Labs** | | | | | | |
| RF30 | Virology Specimen Tracking Form | X | X | X | X | |
| RF31 | Specimen Shipping Form | X | Every odd cycle | X<br>3rd Month | X<br>3rd Month | |
| RF36 | Specimen Procurement Form | X | X | X | X | |
| RF37 | ACSB Procurement Form | X | | | | |
| **To be completed as needed** | | | | | | |
| RF34 | Comments Form | | | | | X |
| RF35 | Missed Visits Form | | | | | X |
| RF04 | Death Form | | | | | X |
| RF05B | Off Protocol Treatment Summary | | | | | X |
| RF50 | Off Study Summary | | | | | X |

[1] Every odd number cycle

[2] Every third month of maintenance or follow-up

## APPENDIX VIII: ACSB INFORMED CONSENT

**INFORMED CONSENT FORM**
**RESEARCH STUDY**
**AIDS MALIGNANCY BANK HIV-RELATED MALIGNANCY**
**TISSUE/BIOLOGICAL FLUIDS BANK**

INTRODUCTION

I am being asked to donate tissue for research. Before I decide to be a part of this research study, I need to understand the risks and benefits so that I can make an informed decision. This is known as informed consent.

This consent form provides information about the research study which has been explained to me. Once I understand the study and the tests it requires, I will be asked to sign this form if I want to take part in the study. My decision to take part in the study is voluntary. This means that I am free to choose if I will take part in the study. Approximately 50 patients will be asked to take part in the study.

PURPOSE

The National Cancer Institute has set up a Bank for tissues and biological fluids from HIV-positive and HIV-negative individuals in order to have specimens available for scientists studying malignancies associated with HIV disease. Individuals who have had biopsies to determine a malignancy are being asked for permission to take some of the tissue for the Bank. Only tissue in excess of that required for decision making will be given to the Bank. If it turns out that my physician needs more of my tissue for additional studies, the Bank will release all of my tissue back to my doctor. No additional tissues will be taken from my body for the Bank.

In addition, I am requested to donate some of my blood to the Bank so that scientists will also be able to look for any deviation in these body fluids that may explain the malignancy.

PROCEDURES

I am being asked for consent to place some of the biopsy material in the Tissue Bank. If I agree to allow the Tissue Bank to have some of my tissue, they would also like to:

1) Confidentially obtain some clinical information from my medical records, now and every six months, that could be useful to research investigators. The report of the information retrieved from my medical record that is given to research investigators will not have my name, or include any information which could personally identify me.

2) Obtain some blood for the Bank. Up to 2 tablespoons of blood will be obtained at my next visit to my physician.

If during the course of treatment by my physician, it is necessary to perform any of the following procedures for diagnostic reasons, I will be asked, at that time, to consent to having a portion of that specimen sent to the Bank. These requests will not require me to make any additional visits to my doctor or have any additional specimens taken just for the Bank. The Bank will only receive part of my specimen, and only what is in excess. No additional materials will be removed for the purposes of the Bank alone. Samples of interest would include, (but are not limited to):

1. Spinal fluid
2. Airway washes
3. Fluid around lungs and intestines
4. Additional biopsy material

I will not be asked to fill out any forms for any of these specimens.

POSSIBLE RISKS

There is a possibility of a bruise and slight pain at the time the blood samples are taken. There is also the possibility of fainting and infection at the site of the blood draw.

POSSIBLE BENEFITS

It may be that there will be no direct benefit to me by consenting to allow the Bank to have portions of my biopsies and biological fluids. However, there may be possible benefits to medical knowledge and other HIV-infected individuals in the future.

COSTS

There will not be any additional costs to me for consenting to participate in this HIV-related malignancy tissue/biological fluids Bank.

PAYMENT FOR INJURY OR HARM

As the list of risks shows, taking part in this research study may result in injury or harm to me. If I require immediate medical care, I should go to an emergency room. Otherwise, the doctor in charge of the study will take care of me or help me get the care you need. I will be sent a bill for whatever medical care I receive. All or part of my bill may be paid by the sponsor of the research study (according to its agreement with the AIDS Associated Malignancies Clinical Trials Consortium), or by my health insurance. This institution will not pay for the care. Likewise, this institution will not pay me for pain, worry, lost income, or non-medical care costs that might occur from taking part in this research study.

PRIVACY

My hospital medical records will be confidentially reviewed to obtain clinical information that could be useful to research investigators. However, the report of this information will not have

my name or social security number anywhere on the report, so I will not be easily identified. The results of this research study will be given to the sponsor, the National Cancer Institute, and may be asked for by the Department of Health and Human Services. In addition, the Institutional Review Board may see my records. Except for these people, records from this study will be kept private unless I authorize their release or release is required by law (i.e. court subpoena). Any publications of this study will not use my name, identify me personally, or include any information which could personally identify me.

QUESTIONS

If I have any questions about this research study, I should contact (Investigator). (Research Nurse or Investigator) _____________ has described to me what is going to be done, the risks, hazards, and benefits involved, and will be available for questions at (institution's number). I understand that my decision to participate or not to participate in this study will not alter my usual health care. In the use of information generated from this study, such as publications, my identity will remain anonymous. The records of studies involving treatment must be available to the FDA and the sponsor and my identity may become known to them. I am aware that I may withdraw from this study at any time without consequence. I further understand that in the event of physical injury or illness occurring to me as a result of the research procedures, care is available but (Institution) will not provide free medical care or compensation for lost wages. Further information with respect to illness or injury resulting from a research procedure as well as a research subjects' rights is available from the Office of the Chief of Staff at (local institution). I understand that by signing this consent form, I do not waive any of my legal rights nor does it relieve investigators or suppliers of liability, but merely indicates that I have been informed about the research study in which I am voluntarily agreeing to participate. A signed copy of this form will be provided to me.

Signature ________________________________ Age ____________ Date____________

________________________________________________________ Date____________
Parent or Guardian signature (if patient is a minor or legally incompetent)

Witnessed by ____________________________________________ Date_____________

Signature of Principal Investigator
________________________ _______________________________Date______________

## APPENDIX IX: ACSB SPECIMEN PREPARATION & SHIPPING INSTRUCTIONS

### COLLECTION

Consent patient for ACSB donation. Collect **20 cc's** approximately 2 tablespoons of whole blood in ACD tubes**.** Fill out the ACSB Specimen Submission form (available on the AMC website under forms and Resources).

### SHIPPING

Fax shipping form to Dr. Sylvia Silver c/o Judith Horn at 202/994-5056.

To ship bloods, place the tubes into a canister of a STP-100 SAF-T-PAK shipper (VWR# 11217-163) wrapping each tube in bubble wrap and using the absorbent paper at the bottom of the canister. Each sample tube should be labeled using a sharpie pen (permanent marker) with the following information:

**Labeling**

- Protocol #: AMC 033
- 9 digit Patient #
- Patient initials
- Date and time of collection
- Specimen type, i.e., WB=Whole Blood, P=Plasma, S=Serum,
- Specimen purpose: Donation

Place the lid on the canister and place it inside of the ambient SAF-T-PAK shipper. **Fold and pack ACSB form inside shipping box.** Seal the ambient shipper with cellophane shipping tape. Label the ambient shipper with the "Infectious substance" diamond shaped label. On one side, in black marker write " INFECTIOUS SUBSTANCE AFFECTING HUMANS (HIV) UN2814, your name or name of responsible person, date of collection and 24 hour phone number of the person responsible for the package.

**Specimen Shipment.** Specimens are accepted MONDAY through THURSDAY. All specimens should be shipped by **overnight express** at room temperature to:

Dr. Sylvia Silver
George Washington University Medical Center
2300 I Street, NW
Room 502
Washington, DC 20037
Phone: (202) 994-1444
Fax: (202) 994-5056

**Instructions for specimens collected on Friday:**

PREPARATION OF PLASMA AND MONONUCLEAR CELLS

It is preferable that separation occurs as soon as possible. If necessary, whole blood in acid citrate dextrose (yellow top tube) can be held at room temperature for no more than 24 hours.

Materials:
Lymphocyte Separation Medium (LSM Solution, Ficoll-Hypaque - sterile)
15 ml conical centrifuge tubes (sterile)
PBS (sterile)
1, 5 ml and 10 ml serologic pipettes (sterile)
NUNC tubes
Alcohol-saturated, control rate freezer container
DMSO freezing media:

- 10% DMSO (dimethylsulfoxide)
- 40% fetal calf serum
- 50% RPMI
- 0.2 mm L-glutamine

Use FED-EX "Dangerous Goods" airway bills for shipping. FED-EX account for the shipment: 2075-8461-4. It is only to be used for billing shipment of specimens to the lab where the sample is processed and/or stored. Call FED-EX at1-800-463-3339 and press 0. Ask for customer service "dangerous goods" department. A FED-EX representative will assist in the specific wording required on the airway bills for pick-up and delivery of "Dangerous Goods". Place the completed airway bill marked **"Priority Overnight"** and the typed "Shippers Declaration for Dangerous Goods" on top of the shipper box inside of a plastic FED-EX pouch. ***PLEASE DOUBLE CHECK PACKAGING OF SHIPPER AND DO NOT DEVIATE FROM REQUESTED LABELING.

**Please Note:** The shipper will be mailed back to the AMC site.
The STP-100 SAF-T-PAK shipper (VWR Cat # 11217-163) is a complete kit w/ all trappings, bubble wrap, absorbent paper, labels, everything (but to reuse the shipper, you will need new labels, wrap, etc). There is a refurbishment kit w/ extra b- wrap, absorbent material (STP102) (VWR Cat # 11217-166) enough for 15 mailings.

**Record of Specimens**. At the time the sample is drawn, complete the "Specimen Procurement Form-RF36." This form will identify when the specimen was drawn. In addition, the "Specimen Shipping Log-RF-31" will be completed. These forms should accompany the sample when shipped and copies sent to the AMC Operations Center.

## Appendix X: Data Safety And Monitoring Plan

### Monitoring The Progress Of Trials And The Safety Of Participants

All AMC protocols follow the CTEP guidelines for reporting of adverse events. All adverse events that meet the expedited reporting requirements of NCI are reported to the AMC Operations Center when they are reported to the Investigational Drug Branch (IDB) of the National Cancer Institute (NCI). These events will be referred to as serious adverse events. If NCI holds the IND or no IND is required for a study, the AMC sites report the serious adverse events directly to the AMC Operations Center. If a commercial sponsor holds the IND, the AMC sites report the serious adverse events to the sponsor, and the sponsor reports them to the AMC Operations Center.

The AMC Operations Center provides a listing of serious adverse events to the protocol chair and co-chair(s) for review on a monthly basis. The AMC Operations Center compiles these events in a tabular format and posts them on the AMC Web site. The AMC Web site is accessible to all AMC investigators and co-investigators. It is the responsibility of each site to provide this information to their respective IRBs, if required by their IRB. For blinded studies, the serious adverse events are reviewed and tabulated without treatment assignment.

Accrual summaries for each AMC trial are posted monthly to the AMC Web page. The progress of each AMC trial is reviewed by the appropriate disease-oriented working group on a monthly conference call. For phase I dose escalation trials, dose escalation (or dose de-escalation) is based on the rules in the protocol and the protocol team determines whether these criteria have been met. For phase II trials, stopping the trial for toxicity or efficacy, or suspending enrollment pending observation of responses in a multi-stage phase II trial, is based on meeting criteria stated in the protocol, and the protocol team determines whether these criteria have been met.

For phase III trials, the AMC has formed an independent Data Safety and Monitoring Committee (DSMC). Voting members of the DSMC are physicians, statisticians, and a patient advocate. All voting members are from outside the AMC. Non-voting members are the NCI scientific project officers and an NCI statistician. The AMC Data Safety and Monitoring Committee reviews AMC phase III studies in accordance with the National Cancer Institute's Policy for Data Safety and Monitoring. Confidential reports of all phase III trials are prepared by the AMC Operations Center Statistician. A written report containing the current status of each trial monitored, and when appropriate, any toxicity and outcome data, are sent to DSMC members by the AMC Operations Center allowing sufficient time for DSMC members to review the report prior to the meeting. This report addresses specific toxicity concerns as well as concerns about the conduct of the trial. The report may contain recommendations for consideration by the DSMC concerning whether to close the trial, report the results, or continue accrual or follow up.

**The results of each DSMC meeting are summarized in a formal report sent by the DSMC Chair to the Group Chair and Operations Center within 1 week of the meeting. The DSMC report contains recommendations on whether to close each study reviewed, whether to report the results, and whether to continue accrual or follow-up. A primary**

**recommendation (e.g., continue with no change; recommended or required modification; stop) must be included in the document. The Group Chair is then responsible for notifying the Study Chair and relevant Disease-oriented Working Group Chair before the recommendations of the DSMC are carried out. In the unlikely event that the Study Chair does not concur with the DSMC, then the NCI Division Director or designee must be informed of the reason for the disagreement. The Study Chair, relevant Disease-oriented Working Group Chair, Group Chair, DSMC Chair and NCI Division Director or designee will be responsible for reaching a mutually acceptable decision about the study. CTEP approval of a formal amendment will be required prior to any implementation of a change to the study.**

**Following a DSMC meeting, a summary of the serious adverse events reported to the DSMC is posted to the AMC Web page. It is each site's responsibility for conveying this information to its IRB.**

**Plans for assuring compliance with requirements regarding the reporting of adverse events (AE).**

For trials monitored by the NCI's Clinical Data Update System (CDUS), adverse event information is transmitted electronically to NCI on a quarterly basis. For trials monitored by NCI's Clinical Trials Monitoring Service (CTMS), adverse event information is transmitted electronically to NCI every two weeks.

**Plans For Assuring That Any Action Resulting In A Temporary Or Permanent Suspension Of An NCI-Funded Clinical Trial Is Reported To The NCI Grant Program Director Responsible For The Grant**

In the event that termination of the trial or major modification to the protocol is under consideration, the protocol chair will convene the protocol team and disease-oriented working group chair by conference call to discuss the options. For phase I and II trials, the protocol chair also has the option of asking the AMC DSMC to review the study. The AMC Operations Center will inform the CTEP Protocol Information Office (PIO) and the Program Director when studies are temporarily or permanently closed. All amendments to protocols must be approved by the Cancer Treatment and Evaluation Program (CTEP) of the National Cancer Institute (NCI).

**Plans for Assuring Data Accuracy and Protocol Compliance**

All study data collected on an AMC clinical trial undergo a data entry and verification process. The case report forms are scanned into the database as the first data entry, and data from the forms are entered into the database by data entry technicians as the 2nd data entry. The dual databases are compared and inconsistencies resolved before forms are added to the study database. Range checks are placed on each field to eliminate entry of out-of-range values. Edit check programs are run periodically on the database to identify and resolve inconsistencies between forms or data collected at different points in time. AMC Operations Center staff interacts with site staff to resolve any data problems.

In accordance with NCI guidelines, AMC Operations Center site monitors visit the AMC sites to evaluate compliance with regulatory issues, and to review data for specific cases by checking source documents. These reports are sent to the AMC Operations Center which forwards them to the site investigator and to the NCI. In the event that major violations are identified, sites are asked to provide a plan to correct deficiencies within 30 days. If needed, a repeat site visit is conducted. In the event that a site does not correct deficiencies in a pre-determined time frame, the AMC Executive Committee has the option of taking action against the site. Possible actions include, but are not limited to, suspending enrollment of new patients to AMC trials until deficiencies are corrected; recommending a decrease in funding to the site; and requiring specific training for site investigators or staff members
